# 3DDPDs: describing protein dynamics for proteochemometric bioactivity prediction. A case for (mutant) G protein-coupled receptors

**DOI:** 10.1186/s13321-023-00745-5

**Published:** 2023-08-28

**Authors:** Marina Gorostiola González, Remco L. van den Broek, Thomas G. M. Braun, Magdalini Chatzopoulou, Willem Jespers, Adriaan P. IJzerman, Laura H. Heitman, Gerard J. P. van Westen

**Affiliations:** 1 Division of Drug Discovery and Safety, Leiden Academic Centre for Drug Research, Leiden University, Leiden, The Netherlands; 2 ONCODE Institute, Leiden, The Netherlands

**Keywords:** 3DDPD, Protein descriptor, Molecular dynamics, Proteochemometrics, GPCR

## Abstract

Proteochemometric (PCM) modelling is a powerful computational drug discovery tool used in bioactivity prediction of potential drug candidates relying on both chemical and protein information. In PCM features are computed to describe small molecules and proteins, which directly impact the quality of the predictive models. State-of-the-art protein descriptors, however, are calculated from the protein sequence and neglect the dynamic nature of proteins. This dynamic nature can be computationally simulated with molecular dynamics (MD). Here, novel 3D dynamic protein descriptors (3DDPDs) were designed to be applied in bioactivity prediction tasks with PCM models. As a test case, publicly available G protein-coupled receptor (GPCR) MD data from GPCRmd was used. GPCRs are membrane-bound proteins, which are activated by hormones and neurotransmitters, and constitute an important target family for drug discovery. GPCRs exist in different conformational states that allow the transmission of diverse signals and that can be modified by ligand interactions, among other factors. To translate the MD-encoded protein dynamics two types of 3DDPDs were considered: one-hot encoded residue-specific (rs) and embedding-like protein-specific (ps) 3DDPDs. The descriptors were developed by calculating distributions of trajectory coordinates and partial charges, applying dimensionality reduction, and subsequently condensing them into vectors per residue or protein, respectively. 3DDPDs were benchmarked on several PCM tasks against state-of-the-art non-dynamic protein descriptors. Our rs- and ps3DDPDs outperformed non-dynamic descriptors in regression tasks using a temporal split and showed comparable performance with a random split and in all classification tasks. Combinations of non-dynamic descriptors with 3DDPDs did not result in increased performance. Finally, the power of 3DDPDs to capture dynamic fluctuations in mutant GPCRs was explored. The results presented here show the potential of including protein dynamic information on machine learning tasks, specifically bioactivity prediction, and open opportunities for applications in drug discovery, including oncology.

**Supplementary Information:**

The online version contains supplementary material available at 10.1186/s13321-023-00745-5.

## Introduction

Proteins are complex biological units that constitute the basis for cellular function. As such, studying their structure and interaction with the environment is a key aspect of preclinical drug discovery [1]. In computational drug discovery, the information encoded in proteins can be extracted and leveraged for several applications using machine learning [2]. These include, among others, target identification [3], computational mutagenesis [4], protein–protein interaction studies [5, 6], and small molecule-target binding affinity prediction [7, 8]. The latter, also referred to as bioactivity proteochemometric modelling (PCM), is an extension of the widely employed quantitative structure–activity relationship (QSAR) models enriched with protein descriptors [7].

Several types of protein descriptors are available for PCM modelling and similar applications [7–9]. These can be broadly classified between sequence-based and structure-based descriptors. Descriptors derived from the protein sequence include discrete features calculated per residue (one-hot encoding) [10] or protein [11] capturing physicochemical properties or amino acid composition. Additionally, deep learning applications of natural language processing have prompted the generation of protein embeddings from sequences [12]. Structure-based descriptors can be derived from molecular graphs or the protein 3D structure by measuring connectivity, distances, and physicochemical properties among others [8, 9]. Moreover, ligand–protein interaction fingerprints can be derived from protein structures in complex with small molecules [13] or from combinations of ligand and protein descriptors [14].

While the goal of protein descriptors is to capture the full complexity of the protein, they largely fail to depict protein dynamism. At physiological temperatures, proteins exist in an equilibrium of structural conformations, which can be studied experimentally or simulated with Molecular Dynamics (MD) [15]. Changes in metabolite or ligand concentrations, as well as mutations and other structural alterations, can impact protein dynamics [15, 16]. These, in turn, directly influence protein function and interactions [15, 17]. The inclusion of dynamic information in protein descriptors could therefore increase performance in some of the machine learning applications listed above. Positive effects have already been reported in target and functional site identification [18], but this potential is yet to be explored in PCM bioactivity modelling.

G protein-coupled receptors (GPCRs) have extensively been explored as targets in bioactivity prediction, including PCM, due to their biological and therapeutic relevance [19, 20]. GPCRs as a family share a highly conserved structure with seven transmembrane (TM) domains that exists in a dynamic equilibrium between active and inactive conformations [21, 22]. In the last decades, the scientific community has seen an increasing interest in the dynamic aspects of GPCRs, resulting in community efforts such as the GPCRmd database, where curated GPCR MD simulations are publicly available [23]. Simultaneously, GPCR research in the context of oncological therapies is gaining momentum [24], with several in vitro studies showing how cancer-related somatic mutations affect receptor function and/or pharmacological intervention [25–27]. Some of the physiological effects observed in mutants have been associated with changes in receptor dynamics thanks to MD simulations [28].

Here, 3D dynamic protein descriptors (3DDPDs) were developed leveraging atom coordinates and partial charges from publicly available single replicate MD simulations from GPCRmd. Two descriptor architectures were explored: embedding-like (protein specific—ps3DDPD), and one-hot encodings (residue specific—rs3DDPD). The performance in PCM GPCR bioactivity prediction of these novel protein descriptors was benchmarked against and in combination with a panel of state-of-the-art protein descriptors. Finally, the ability of our 3DDPDs to capture dynamic changes driven by (cancer-related) somatic point mutations in GPCRs was tested. These results highlight 3DDPDs as a stepping stone for further research on protein descriptors used for predicting drug-target interactions based on protein dynamics.

## Results

### 3DDPDs generation and optimization

**Fig. 1.**
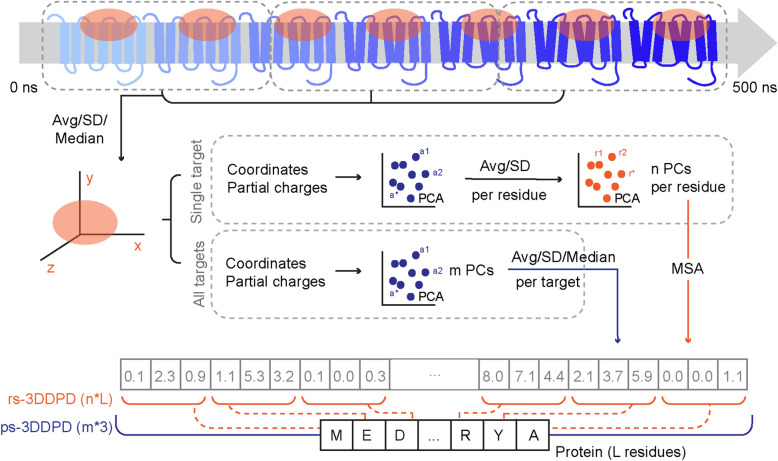
3D dynamic descriptor (3DDPD) generation overview. First, a selection of residues and atoms is made. XYZ coordinates are collected for the selected atoms over all frames of the trajectory. The full simulation ranging from 0 to 500 ns is divided into sub-trajectories and atomic coordinate statistics (average, SD, and median) are computed for each of them. Two routes are possible from this point to generate either one-hot encoded residue-specific rs3DDPDs or embedding-like protein-specific ps3DDPDs. Respectively, atomic data is grouped and standardized either per target or for all targets and PCA is computed. A number of PCs for each atom are then selected and, in the case of rs3DDPDs, grouped per residue by calculating the average and SD. A second dimensionality reduction step is applied to residue data and the selected n number of PCs are mapped to their corresponding positions in a MSA of the targets of interest. This results in a vector rs3DDPD of length n * L, where L is the length of the protein or the MSA. For ps3DDPDs, the m number of PCs selected per atom are grouped per target by calculating average, median, and SD, therefore resulting in the final vector descriptor of length m * 3

3D dynamic protein descriptors (3DDPDs) were designed to capture the dynamic behavior of proteins in MD simulations. For this purpose, atomic coordinates were first extracted from the MD trajectories and their variability over a certain number of frames calculated. As proof of concept, 3DDPDs were conceived for single MD trajectory replicates in this work. In order to account not only for the position but also for the type of atoms in the protein, atomic partial charges were computed. Next, two strategies were developed to condense the dense atomic information into protein descriptors (Fig. 1). These strategies correspond to the two types of 3DDPDs envisioned. The residue-specific (rs)3DDPD is closer to classical one-hot encoded protein descriptors and defines each residue in the protein with a fixed number of features. The rs3DDPD was designed to capture the differences across different sections of the target. The second type, protein-specific (ps)3DDPD, is closer to whole sequence protein embeddings and was designed to capture the differences between targets in a set. Consequently, atomic data were aggregated per target for rs3DDPDs and for all targets for ps3DDPDs and its dimensionality was reduced via principal component analysis (PCA). Several principal components (PCs) for each atom were selected and, in the case of rs3DDPDs, grouped per residue. A second dimensionality reduction step was applied to residue data and the selected PCs were placed in their matching sections corresponding to a multiple sequence alignment (MSA) of the targets of interest. For ps3DDPDs, the PCs selected per atom were grouped per target, resulting in the final descriptor.

The 3DDPD generation strategy described above was optimized by comparing the descriptors’ performance on PCM modelling tasks. GPCRs were selected as the protein family for this case study given the availability of a large number of MD trajectories freely in the GPCRmd database [23]. Particularly, the focus laid on Class A GPCR apo structures in the inactive or intermediate conformations, more broadly represented at the time of the analysis. The PCM dataset contained 26 GPCRs with available MD trajectories in GPCRmd and high-quality data in the Papyrus bioactivity dataset [29], in total 38,701 datapoints. Although two data split strategies (i.e. random and temporal) were applied in both regression and classification PCM tasks, the optimization strategy was driven mostly by the results in the most demanding task, regression with a temporal split.

**Fig. 2:**
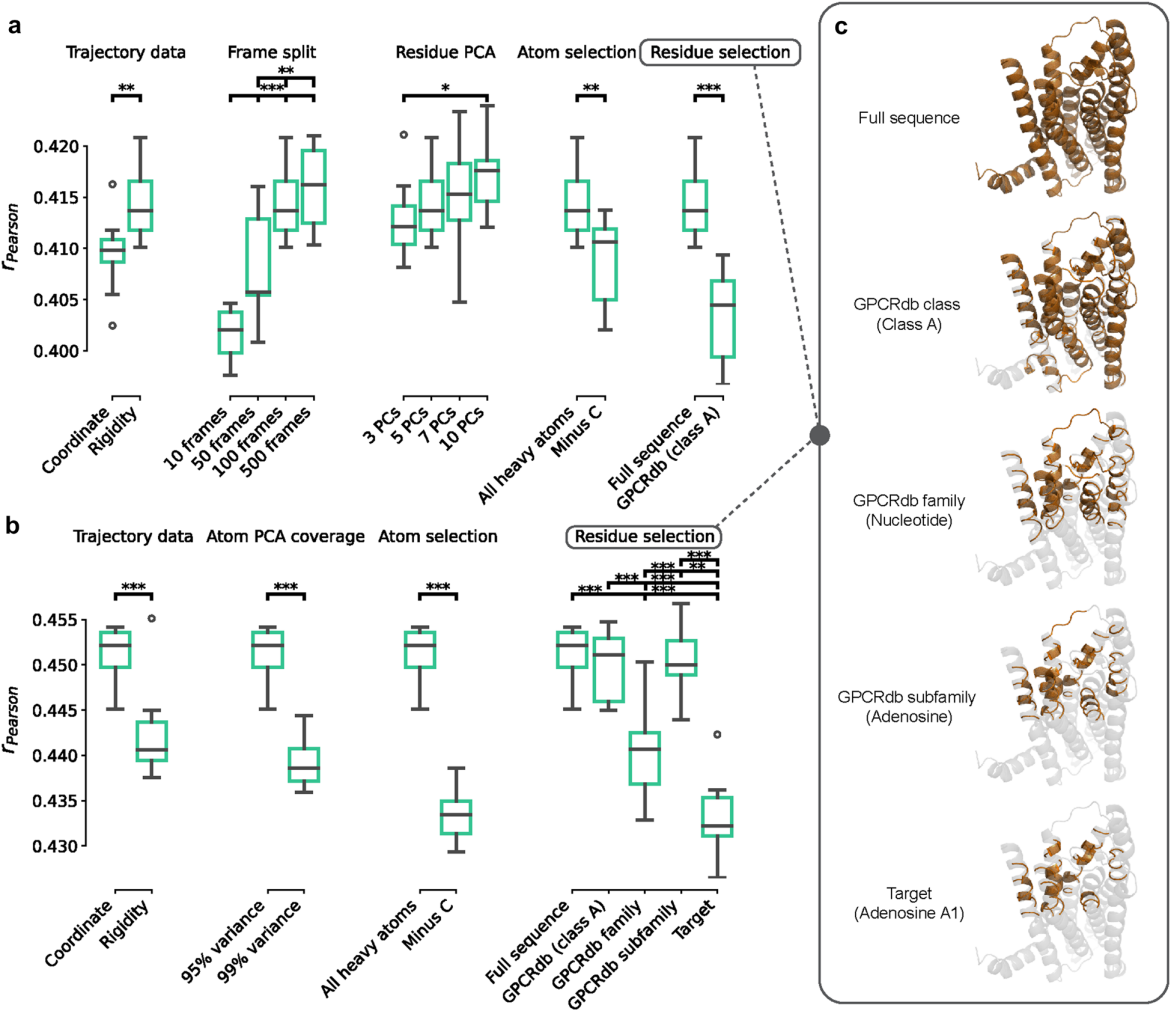
Optimization of the 3DDPD generation strategy. Ten PCM regression tasks with temporal split were trained with each variation of the 3DDPDs to select the optimal parameters. Pairwise differences were analyzed by their statistical significance in a Student’s T test, represented by asterisks in (a,b):. * = p-value < 0.05; ** = p-value < 0.01; *** = p-value < 0.001. **a** rs3DDPDs were optimized by testing different options for trajectory data (i.e. choices of statistical metrics for sub-trajectory grouped coordinate atomic data: “coordinate” includes all, “rigidity” only SD), number of frames in the sub-trajectory frame splits, number of PCs from the residue PCA, atom selection (i.e. all heavy atoms or “minus C”: non-carbon), and residue selection (i.e. full sequence or class A GPCRdb-annotated binding pocket). **b** ps3DDPDs were optimized based on trajectory data, variance covered by the selected number of atom PCA components, atom selection, and residue selection. **c** Residue selection options exemplified on the structure of adenosine A1 receptor PDB 5UEN. In orange, the residues that would be selected by each of the five possible definitions of a structural-driven binding pocket selection approach: full sequence, class A, family, subfamily, and target

First, the “dynamic” properties derived from atomic coordinates were optimized. Here, the use of mean, median, and standard deviation from the mean (SD) or just the SD, representing the “rigidity” of each atomic coordinate was benchmarked. For rs3DDPDs, using SD resulted in better performance (Fig. 2a), contrary to ps3DDPDs (Fig. 2b). The number of frames included in each trajectory split was also optimized, where 100 or 500 frames yielded similarly better results (Fig. 2a), so 100 frames were selected further. The variance explained by the selected number of PCs on atom data was optimized and set at 95% for both rs3DDPDs and ps3DDPDs (Fig. 2b), and similarly, the number of PCs on residue data was optimized and set to 5 not to explode the number of features (Fig. 2a).

Furthermore, the inclusion of atomic data from all heavy atoms or non-carbon atoms only was tested. The former option was significantly better for both rs3DDPDs (Fig. 2a) and ps3DDPDs. Finally, residue selection strategies were tested to focus the 3DDPDs on the protein binding site (Fig. 2c). These selections were based on structural-driven MSAs at different protein family levels, starting from the full sequence, then the binding pocket of class A GPCRs, then specific GPCR families, such as nucleotide receptors, then GPCR subfamilies, such as adenosine receptors, and finally, target-specific binding pocket such as the adenosine A_1_ receptor. To ensure a consistent number of features per descriptor, in rs3DDPDs only the first two options could be tested, where the class A binding pocket performed significantly worse than the full sequence (Fig. 2a). In ps3DDPDs all selection methods performed similarly except for the family and target pockets, which performed significantly worse (Fig. 2b).

The optimized rs3DDPD included “Rigidity” coordinate data calculated from 100-frame splits, where all atomic data was included for all residues in the protein sequence. In the atomic PCA, 95% of the variability was kept and 5 PCs in the residue PCA. This resulted in a vector of 3,785 features for the class A GPCRdb MSA used, of length 757. The optimized ps3DDPD included all coordinate data statistics calculated from 100-frame splits, where all atomic data was included for all residues in the protein sequence and 95% of the variability was kept in the atomic PCA. This resulted in a vector of 30 features.

### 3DDPDs reflect the GPCR dynamic fluctuations

**Fig. 3:**
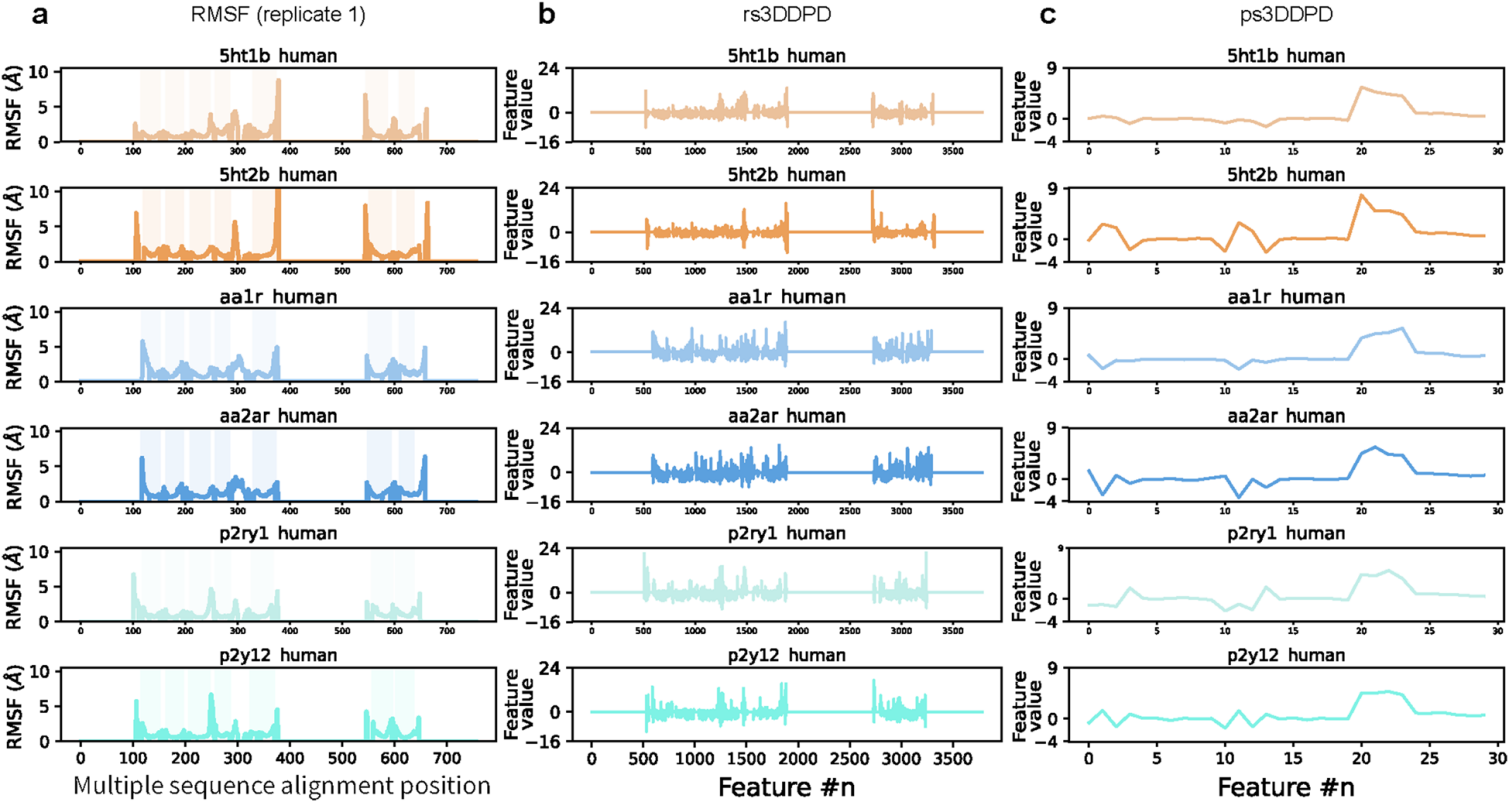
Representation of the GPCRs dynamic behavior by 3DDPDs. **a** Dynamic fluctuations of the residues of six GPCRs from the set, represented by their RMSF (Å). The RMSF values are mapped to their corresponding positions in the MSA later used for rs3DDPD and non-dynamic descriptor calculation, for easier visualization. The regions in the MSA corresponding to domains TM 1–7 are shadowed for reference. Data for the complete set of 26 GPCRs is available in Additional file 2: Fig S1. **b** Representation of the rs3DDPD feature values for the same subset of GPCRs. Data for the complete set of 26 GPCRs is available in Additional file 2: Fig S3. **c** Representation of the ps3DDPD feature values for the same subset of GPCRs. Data for the complete set of 26 GPCRs is available in Additional file 2: Fig S4

From the publicly available MD database for GPCRs, GPCRmd, a subset of 26 trajectories for class A GPCRs with sufficient bioactivity data for PCM modelling was selected, as described in the *Materials and Methods* section. Apo inactive conformations were selected to avoid bias towards a specific ligand-triggering activation mode. The targets selected covered 17 subfamilies within four class A families: aminergic, lipid, nucleotide, and peptide receptors. The analysis of the MD trajectories showed similarities between dynamic behaviors but also differences that can be potentially captured and exploited using 3DDPDs. Such differences can be better observed by aligning the Root Mean Square Fluctuation (RMSF) values to a GPCR class A MSA (Fig. 3a and Additional file 2: Fig S1). Across GPCRs, there is a shared pattern of reduced mobility in the TM domains compared to extracellular (ECL) and intracellular (ICL) loops or N- and C-terminus. However, deviations from this pattern are common when comparing (i) members of different families (e.g. adrenergic 5-hydroxytryptamine receptor 1_B_ (5HT1B) and nucleotide adenosine A_1_ receptor (AA1R) in their overall dynamic behavior), (ii) members of the same family but different subfamilies (e.g. nucleotide receptors adenosine A_2A_ (AA2AR) and P2Y purinoceptor 1 (P2RY1) in TM2, ICL2, ECL2, ICL3, and C-terminus), or (iii) even members of the same subfamily (e.g. 5-hydroxytryptamine receptors 5HT1B and 2_B_ (5HT2B) in N-terminus, TM3, TM4, ECL2, ICL3, and ECL3). Importantly, the main dynamic patterns described above were highly conserved for the three different replicates of the same system available on GPCRmd (Additional file 2: Fig S2), suggesting that the omission of MD replicates in the current 3DDPD pipeline did not have a major impact on the results presented here.

The observed similarities and differences in dynamic behaviors between GPCRs were effectively captured by the optimized rs3DDPDs (Fig. 3b and Additional file 2: Fig S3) and ps3DDPDs (Fig. 3c and Additional file 2: Fig S4). In the translation from RMSF to rs3DDPD and ps3DDPD, positive and negative values appeared that represented inter- and intra-target variability, respectively. While rs3DDPDs reflected the dynamic fluctuations on a residue level that resembled more closely the RMSF pattern itself, ps3DDPDs showed a more generalized embedding of each protein dynamics compared to all the targets in the set thus enhancing the differences among targets. Of note, rs3DDPDs did not represent merely a transform of the RMSF values, as exemplified for the positions corresponding to the N-terminus and TM1 in P2RY1 and P2RY12 (Fig. 3a, b). This suggests that information other than the atom coordinate variability, such as the type of atoms and residues encoded by partial charges, was picked up by the 3DDPDs. In part, such an effect was likely possible thanks to the dimensionality reduction process that introduced several opportunities to exploit atomic and residue similarities and differences as opposed to the RMSF calculation.

### 3DDPDs outperform non-dynamic protein descriptors in PCM regression tasks

The use of 3DDPDs as protein descriptors in PCM bioactivity modelling tasks was tested for our GPCR dataset. For this purpose, the performance of random forest (RF) models was benchmarked using 3DDPDs in combination with ECFP6 molecular fingerprints against models using as protein descriptors one of five other one-hot encoded descriptors (i.e. Zscale in two modalities, STscale, MS-WHIM, and PhysChem) or one protein embedding (i.e. Unirep). The benchmark was carried out for classification and regression tasks using two different types of training-test splits: 80:20 random split and temporal split with 2013 as a cutoff year for the test set. The temporal split was introduced as a more accurate representation of a drug discovery campaign where data from the past is used to predict novel chemical entities developed later in time and indeed showed a considerable decrease in chemical bias compared to the random split (0.051 vs. 0.279).

The bioactivity dataset compiled for bioactivity modelling contained 38,701 bioactivity datapoints heterogeneously distributed across the 26 targets (Additional file 1: Table S1). Active data for classification was defined with a cutoff of 6.5 pChEMBL value. Firstly, the need for PCM modelling in such a set was assessed by comparing the performance of the PCM models to the average performance of individual QSAR models for each of the GPCRs in the set. In all of the modelling scenarios, the worst performing PCM model outperformed significantly the QSAR models: Matthews correlation coefficient (MCC) 0.643 ± 0.005 (UniRep) vs. 0.578 ± 0.007 in random split classification, MCC 0.273 ± 0.003 (rs3DDPD) vs. 0.192 ± 0.009 in temporal split classification, Pearson r 0.832 ± 0.003 (UniRep) vs. 0.775 ± 0.005 in random split regression, and Pearson r 0.410 ± 0.003 (Zscale Hellberg) vs. 0.343 ± 0.004 in temporal split regression.

**Fig. 4:**
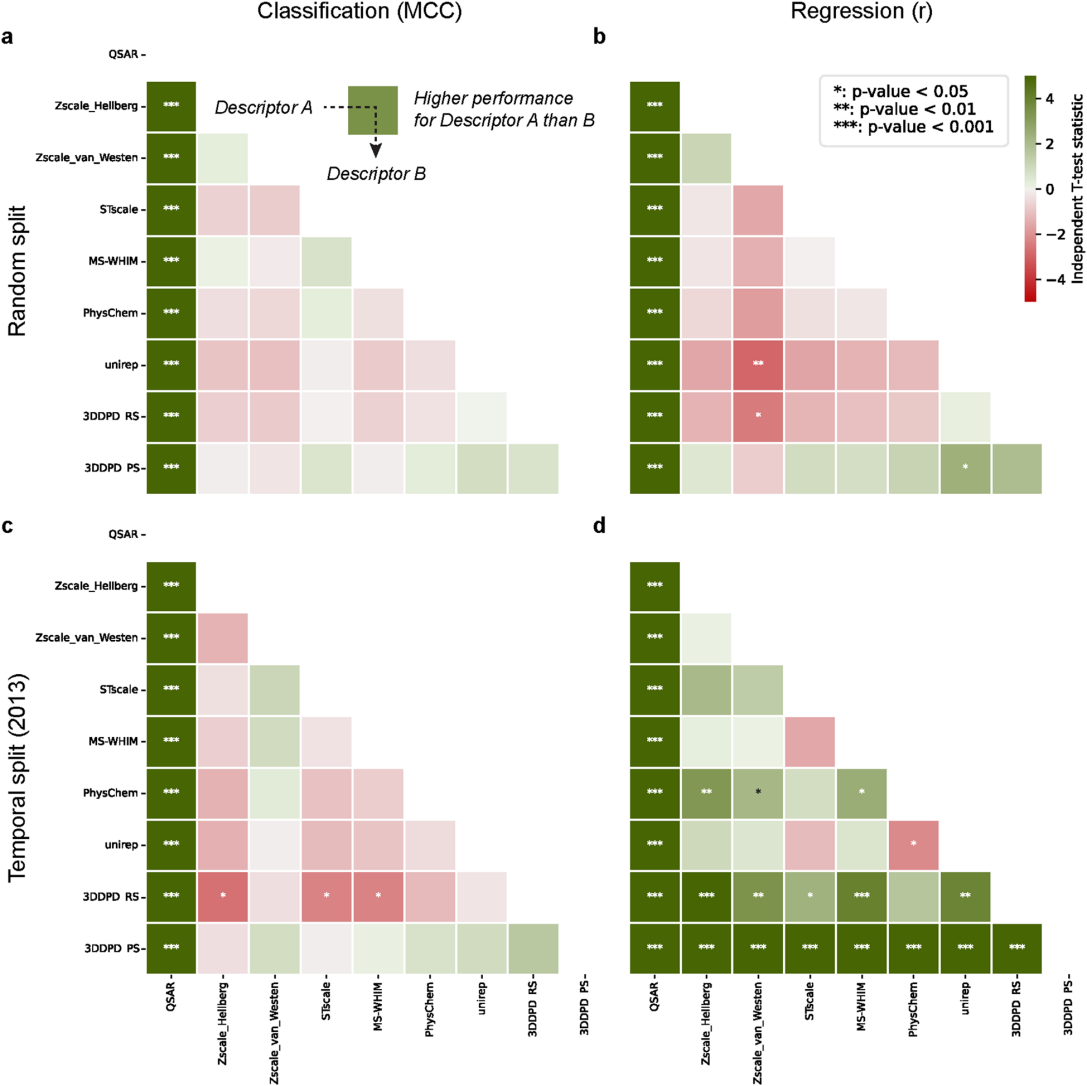
Benchmark of 3DDPD performance in PCM bioactivity modelling tasks against non-dynamic descriptors. Ten RF models with random seeds were trained and validated for each combination of protein descriptors with ECFP6 molecular fingerprints. A shade of green (the darker the better) represents better performance using a descriptor A instead of a descriptor B, as read in panel a. A shade of red (the darker the worse) represents worse performance using a descriptor A instead of a descriptor B. The statistical significance of the differences is derived from pairwise Student T-test and represented by asterisks: * = p-value < 0.05; ** = p-value < 0.01; *** = p-value < 0.001. Four PCM tasks were benchmarked: **a** Classification with validation based on an 80:20 random split. In classification tasks, MCC was used as an evaluation metric on the test set. **b** Regression with validation based on 80:20 random split. In regression tasks, Pearson r was used as an evaluation metric on the test set. **c** Classification with validation based on a temporal split, with 2013 as the cutoff year. **d** Regression with validation based on a temporal split, with 2013 as the cutoff year

In PCM, models using 3DDPDs performed similarly to using other protein descriptors in classification tasks regardless of the split type (Fig. 4a, c). One exception was the temporal split classification task, here rs3DDPDs produced slightly worse performance than models using Zscale Hellberg, Stscale, and MS-WHIM (MCC 0.273 ± 0.003 vs. 0.273 ± 0.005, 0.278 ± 0.005 and 0.277 ± 0.004, respectively, Fig. 4c). In the regression task with random split, models using 3DDPDs performed again similarly to models using other protein descriptors (Fig. 4b), with the exception of rs3DDPDs performing slightly but significantly worse than Zscale van Westen (Pearson r 0.832 ± 0.004 vs. 0.836 ± 0.004, respectively) and ps3DDPDs performing slightly better than the Unirep protein embedding (Pearson r 0.835 ± 0.003 vs. 0.832 ± 0.003, respectively). In the regression task with temporal split, however, both types of 3DDPDs outperformed the rest of the descriptors (Fig. 4d). The performance of models trained with non-dynamic protein descriptors measured as Pearson r ranged from 0.410 ± 0.003 (Zscale Hellberg) to 0.415 ± 0.004 (PhysChem) passing by 0.410 ± 0.006 (Zscale van Westen), 0.410 ± 0.004 (MS-WHIM), 0.411 ± 0.004 (UniRep), and 0.413 ± 0.005 (Stscale). One-hot encoded rs3DDPDs performed significantly better than most of the other descriptors, except for PhysChem, with a Pearson r of 0.417 ± 0.004. Embedding-like ps3DDPDs, however, significantly outperformed all the other descriptors, including rs3DDPDs, with a Pearson r of 0.451 ± 0.003. These results were also confirmed in terms of Root Mean Square Error (RMSE), which was the lowest for ps3DDPDs (1.154 ± 0.003) and then QSAR models on average (1.168 ± 0.004), followed by rs3DDPDs (1.214 ± 0.005) and then the rest of non-dynamic protein descriptors (from 1.124 ± 0.005 to 1.221 ± 0.006). A summary of all validation metrics is given in Additional file 1: Table S2 (random split) and Additional file 1: Table S3 (temporal split).

**Fig. 5:**
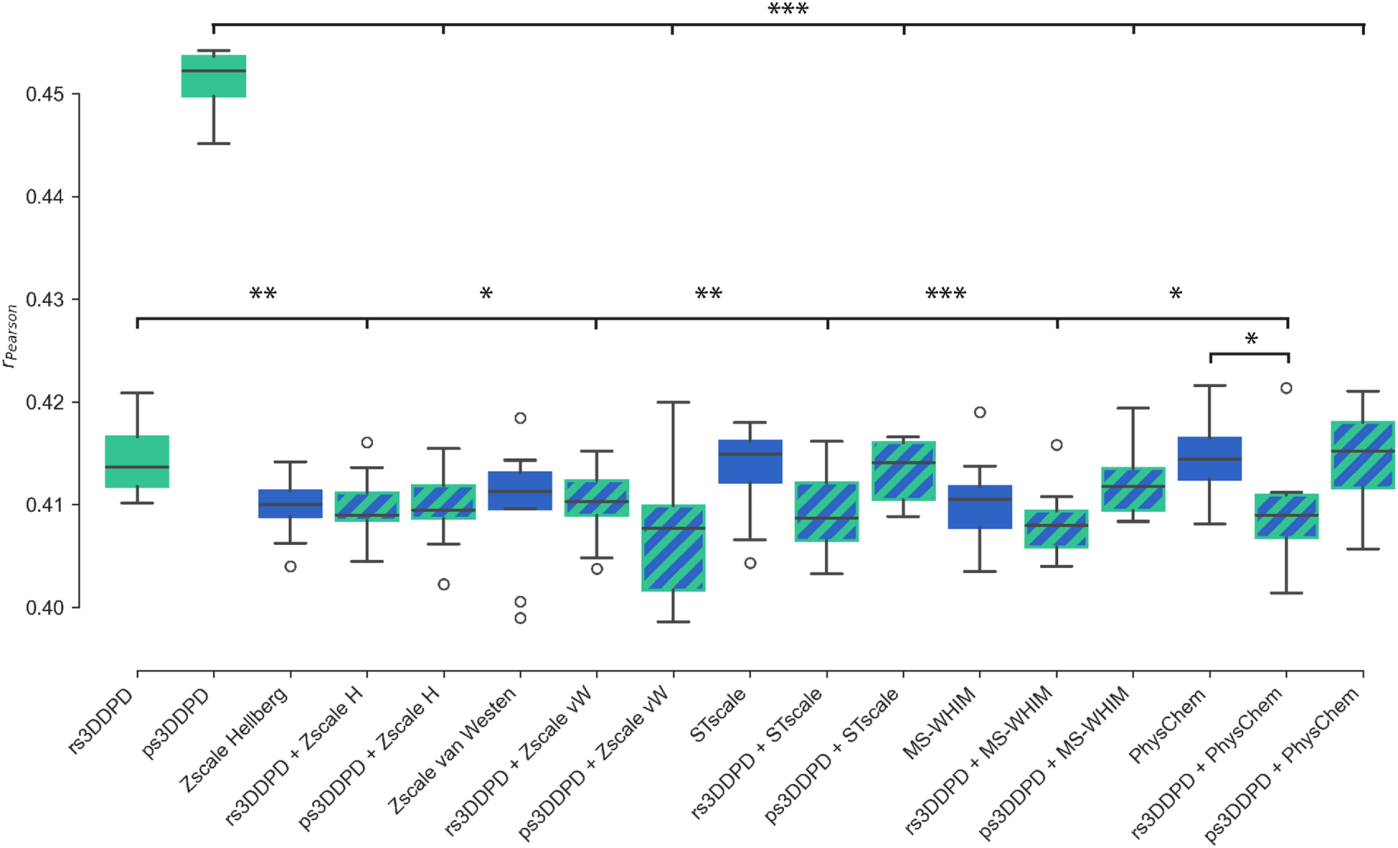
PCM model performance with dynamic and non-dynamic protein descriptor combination in regression tasks with a temporal split. In green, the performance of RF models trained on 3DDPDs. In blue, RF models trained on non-dynamic protein descriptors. In green and blue, RF models trained on a combination of both types. Zscale Hellberg and van Westen are abbreviated to Zscale H and vW, respectively. The statistical significance of the differences is derived from pairwise Student T-test and represented by asterisks: * = p-value < 0.05; ** = p-value < 0.01; *** = p-value < 0.001

In order to test the complementarity of the 3DDPDs with other protein descriptors, a set of regression models was trained with temporal splits with pairs of dynamic and non-dynamic protein descriptors (Fig. 5). In all cases, the addition of a 3DDPD on top of a non-dynamic descriptor resulted in similar performance to the models trained exclusively using non-dynamic descriptors, or even slightly worse in the case of PhysChem + rs3DDPD. Moreover, the combination yielded statistically worse performance than using the dynamic descriptors alone, particularly in the case of ps3DDPD. This non-complementarity was further confirmed for ps3DDPDs by their exclusion from the most important features for the combination models (e.g. ps3DDPD + PhysChem, Additional file 2: Fig S5d), where only non-dynamic protein descriptor features and ECFP6 compound fingerprint bits were picked up as the top 25 most important for the model. For rs3DDPDs, however, there seemed to be a certain complementarity as both dynamic and non-dynamic protein descriptor features showed up among the top 25 most important for the model (e.g. rs3DDPD + Zscale van Westen, Additional file 2: Fig S5c), even if this did not translate into an improvement in model performance.

### rs3DDPD features can be traced back to generic GPCR positions

A specific trait of one-hot encoded protein descriptors is that every feature can be traced back to specific protein sequence residues or MSA positions. For class A GPCRs, the aligned positions can additionally be linked to generic positions in the GPCR structure with known functional relevance. The most widely used generic position identifier for class A GPCRs is the Ballesteros-Weinstein (BW) schema [30], which consists of a first number identifying the TM domain followed by a second number that represents the level of conservation in that helix around the most conserved position that gets the value 50. Using the GPCRdb [31] MSA mapping to BW positions, the most important rs3DDPD features in regression models were traced back to their generic GPCR positions.

**Fig. 6:**
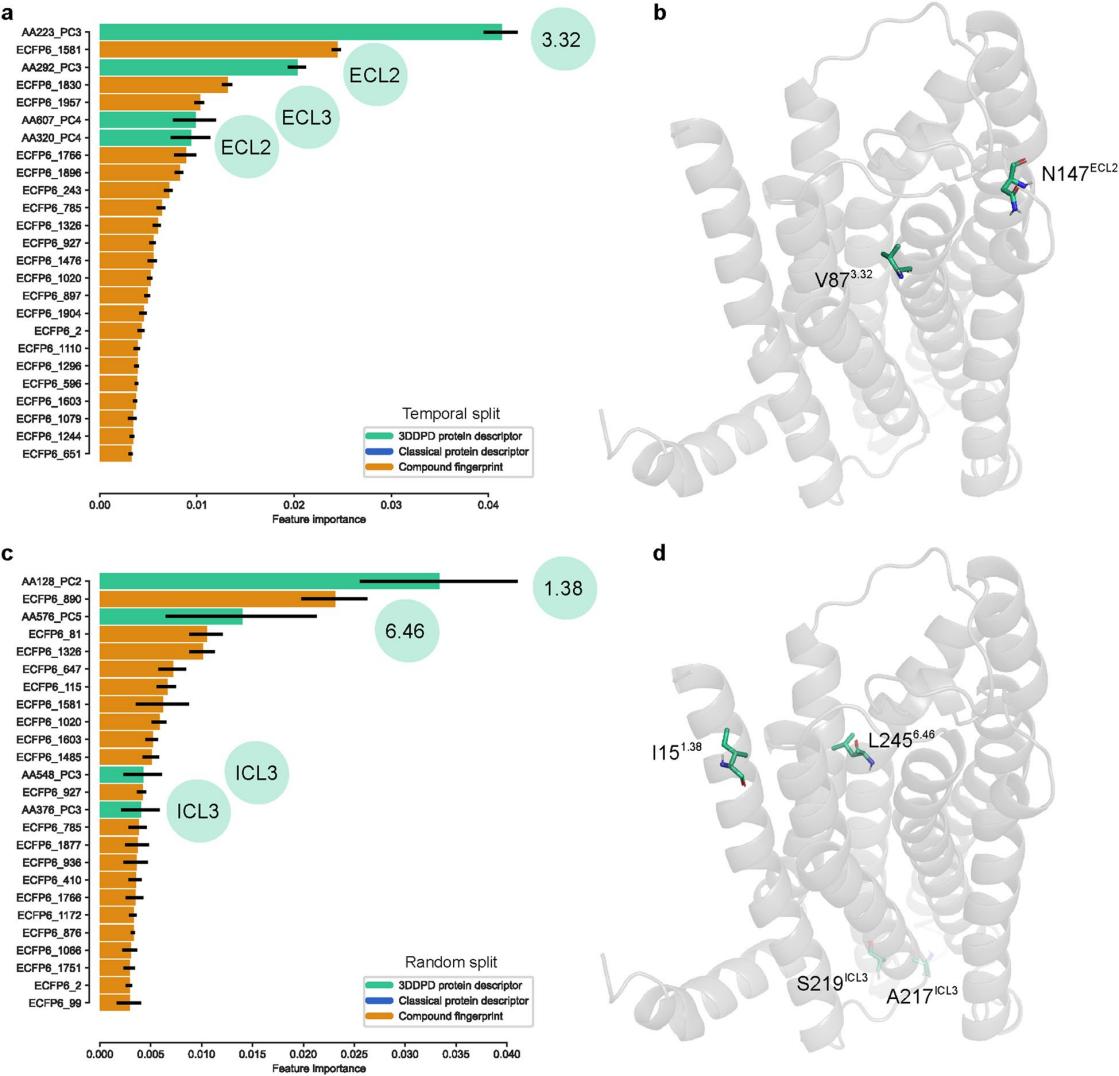
GPCR generic position mapping of most important rs3DDPD features in PCM regression tasks. **a** Top 25 most important features in PCM regression models using a temporal split validation for the GPCR set. The importance was averaged across the ten random seeds trained and the SD represented as error bars. Rs3DDPD features are mapped to their corresponding GPCR Ballesteros-Weinstein number or, if not available, region of the protein. **b** Representation of the most important rs3DDPD features in regression temporal split in the adenosine A1 receptor (PDB 5UEN). **c** Top 25 most important features in PCM regression models using a random split validation. **d** Representation of the most important rs3DDPD features in regression random split in the adenosine A1 receptor)

In the models built with a temporal split, four rs3DDPD features were among the top 25 most important (Fig. 6a). The most important feature overall, *AA223_PC3*, corresponded to the BW position 3.32 in TM3. For further interpretability, this generic position can also be directly mapped to a specific residue in a protein of interest. As an example, in AA1R 3.32 it translated to Val 87 (Fig. 6b). The other three important rs3DDPD features did not correspond to any BW positions, as two of them were located in the ECL2 and one in the ECL3. From the three loop positions, only one exists in adenosine receptor A1, Asn 147 (*AA292_PC3*). The two other ECL positions are only available in other receptors (Additional file 2: Fig S1). In the models built with a random split, the two most important rs3DDPD features, *AA128_PC2* and *AA576_PC5*, corresponded to TM1 1.38 and TM6 6.46 BW positions, respectively (Fig. 6c). In AA1R, these translated to Ile 15 and Leu 245 (Fig. 6d). The other two important rs3DDPD features correspond to positions in ICL3. Of note, the consensus between seeds on the importance of specific rs3DDPD features was less marked on the models with random split than on the models with temporal split (Fig. 6a, c). This analysis was further applied to discuss the relevance of specific GPCR positions in ligand binding.

### Dynamic fluctuations in mutants can be captured with 3DDPDs

To assess the viability of dynamic descriptors to capture differences between mutants in a potential mutant PCM model, a subset of 28 mutants from five of the GPCRs in our set was gathered: AA1R and AA2AR, muscarinic acetylcholine receptor 2 (ACM2), beta-2 adrenergic receptor (ADRB2), and CC chemokine receptor 5 (CCR5). The selection of mutations was done for the original set of 26 GPCRs when there was available mutagenesis data in GPCRdb (Table 2), from which the point mutation’s effect in bioactivity was projected for the five resulting receptors (Additional file 2: Fig S6). Additionally, five mutations on these GPCRs present in cancer patients from the Genomic Data Commons (GDC) database were included that also had mutagenesis data in GPCRdb: AA1R R291C^7.56^ and R296C^8.51^, AA2AR H278N^7.42^, ACM2 V421L^7.33^, and ADRB2 V317A^7.43^. The cancer-related mutants, however, did not seem to have an effect on bioactivity given the limited amount of mutagenesis data available.

The selected mutations were introduced in equilibrated wild type receptor systems from GPCRmd, which were subsequently re-equilibrated to run production 500 ns MD simulations following the GPCRmd pipeline. One of the selected mutations did not run successfully therefore it was discarded from the analysis (AA2AR H278N^7.42)^. Most mutant trajectories showed deviations from wild type trajectories in terms of RMSF (Additional file 2: Fig S7), with the exception of AA1R and CCR5 mutants. The deviations were sometimes in the vicinity of the mutation (i.e. AA2AR M177A^5.40^, N181A^5.43^, Y271A^7.35^; ARDB2 D130N^3.49^, S203A^5.43^, V317A^7.43^; ACM2 D103E^3.32^, V421L^7.33^), but most commonly spawned across the whole sequence or altered stability in distant regions. For example, in AA2AR L85A^3.33^ increased flexibility in ICL2 and ECL2 and S91A^3.39^ in ICL3 and TM6. Moreover, adjacent mutations that triggered different effects were observed. For example, in ADRB2, S203A^5.43^ decreased stability in TM1, ICL2, and ECL3, while S204A^5.44^ decreased stability in TM2 and TM4 while increasing stability in ICL3. Of note, in ACM2 D103E^3.32^ and D103N^3.32^ triggered similar higher flexibility in ECL1 and ECL2, with an overall differential pattern of lower stability in D103E^3.32^. In general, the mutations with smaller dynamic fluctuations from the wild type also corresponded to those with a smaller effect in bioactivity, such as AA1R R291C^7.56^ and R296C^8.51^, and ADRB2 V317A^7.43^ (Additional file 2: Figs S6, S7).

**Fig. 7:**
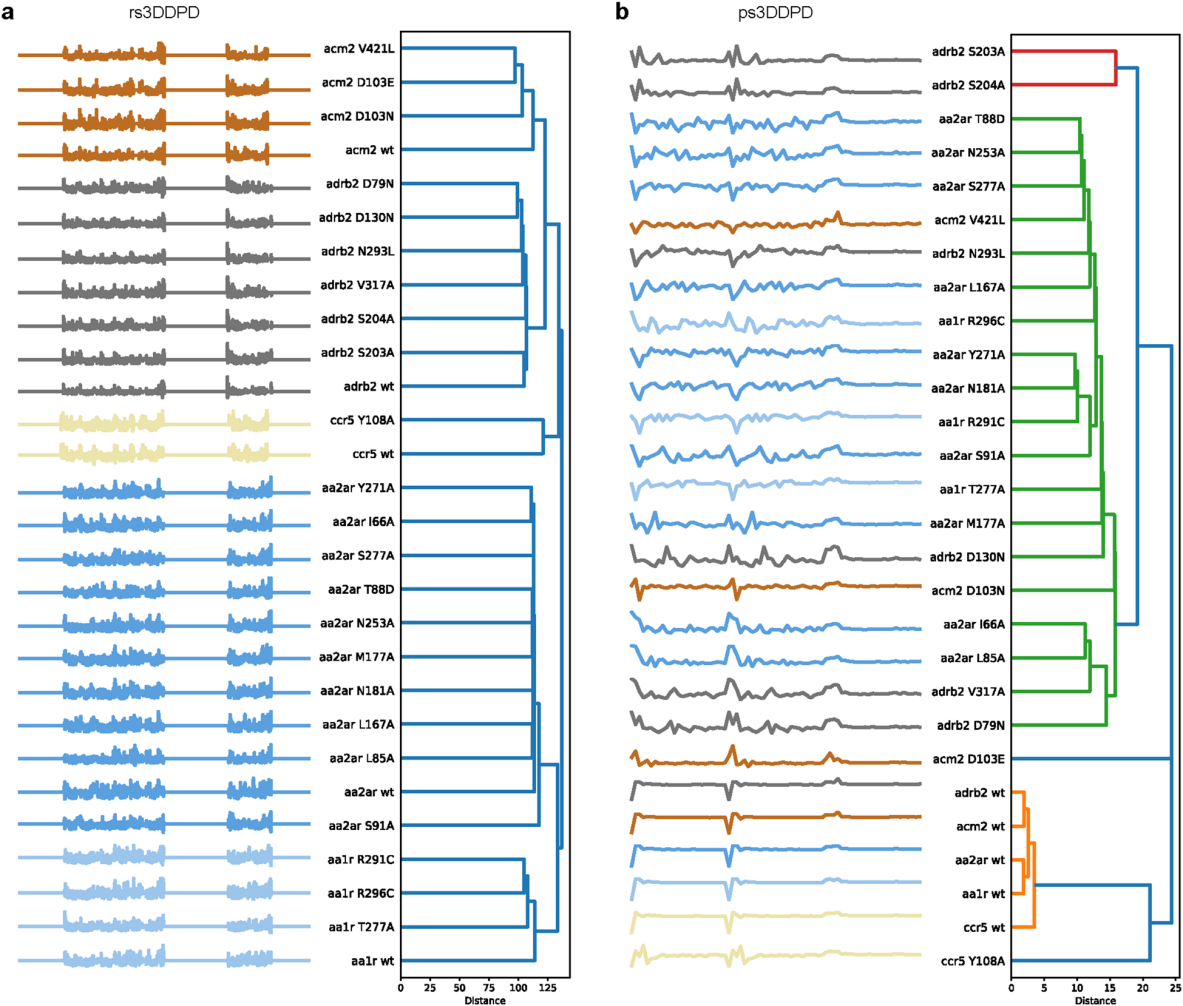
Discrimination of GPCR mutants using 3DDPDs as descriptors. Hierarchical clustering of GPCR variants based on their Euclidean distance between descriptor vectors. **a** Mutants represented as rs3DDPDs. **b** Mutants represented as ps3DDPDs. Individual clusters generated under a distance threshold of 70% of the final merge are represented in different colors in the dendrograms

Next, the power of 3DDPDs to distinguish between mutants was tested. rs3DDPDs and ps3DDPDs were computed for the mutant trajectories and used to cluster the mutants based on the distance between descriptors. As rs3DDPDs are computed independently for each trajectory and reflect all atoms in the system, all mutants of the same target clustered together (Fig. 7a). Within targets, clusters of mutants with similar overall dynamic behavior compared to wild type were observed, for example, ADRB2 D79N^2.50^ and D130N^3.49^, or with similar fluctuations from wild type in specific regions, such as AA1R R291C^7.56^ and R296C^8.51^ in TM7 and H8/C-terminus (Additional file 2: Fig S7). For targets with unique differential dynamic patterns from wild type for each mutant, like ACM2, the clusters discerned the most different patterns (e.g. D103N^3.32^ shows certain receptor stabilization compared to D103E^3.32^ and V421L^7.33^, and is therefore excluded from the cluster). These results supported the ability of rs3DDPDs to capture dynamic fluctuations in mutants. Nevertheless, the mutant discriminatory power of rs3DDPDs did not correlate directly to that of using directly RMSF (Additional file 2: Fig S8a) or RMSF differences to wild type (Additional file 2: Fig S8b), which reinforced the notion that rs3DDPDs are not merely a transform of RMSF and include other non-dynamic atomic information.

Using ps3DDPDs, mutants were clustered based on overall similarities and differences in their dynamic behavior and residue composition across the set (Fig. 7b). This way, the five wild type targets clustered together because they had the most stable trajectories overall, and CCR5 Y108A^3.32^ was close by because overall it showed small differences to the wild type trajectory (Additional file 2: Fig S7). However, some discrepancies with the expected results based on RMSF differences were found. For example, ADRB2 S203A^5.43^ and S204A^5.44^ formed their own cluster despite showing differential RMSF peaks. This and other examples suggest that ps3DDPD values for this set of mutants were heavily influenced by fluctuations in the N- and C-terminus, which were the most accentuated. Therefore ps3DDPDs did capture mutant fluctuations, but using them in their optimized form for wild type GPCRs seemed suboptimal to discriminate mutants.

## Discussion

PCM is a modality of bioactivity modelling that leverages similarities and differences between targets by combining them in the same model represented by protein descriptors [7]. The most commonly used protein descriptors in PCM characterize different properties of the sequence of residues [10], but do not consider an important factor for protein–ligand binding: protein dynamics. Here, 3D dynamic protein descriptors (3DDPDs) were developed leveraging publicly available single-replicate MD simulations. This information was condensed into multiple steps that were optimized to produce a one-hot encoding residue-specific (rs3DDPD) and an embedding-like protein-specific (ps3DDPD) descriptor. The optimized 3DDPDs were subsequently benchmarked against non-dynamic protein descriptors in PCM tasks for a bioactivity set of 26 class A GPCRs. Finally, the use of 3DDPDs to describe point mutations was explored, which are otherwise underrepresented by sequence-based non-dynamic descriptors.

The strategy to develop 3DDPDs borrows ingredients from other types of descriptors. Firstly the calculation of 3DDPDs starts from the collection of coordinate data for each atom, to which atomic partial charges were added to represent the electrostatic component over time (Fig. 1). Other MD fingerprints for small molecules have used as starting properties potential energy, solvent-accessible surface area or radius of gyration [32], ultimately similarly representing electrostatic and conformational changes of the molecule over time. More computationally expensive partial charges than Gasteiger could be explored, although the simpler implementation chosen here has been shown to be a cost-efficient option in other modelling tasks [33]. Further down in our pipeline, PCA is used to reduce dimensionality, which is a common resource in protein descriptor calculation. However, for non-dynamic one-hot encoded descriptors, it is often used to calculate fixed features for each residue type (e.g. Zscale, MS-WHIM, Stscale [10, 34]) rather than specific features for each residue in the sequence, as was done for rs3DDPDs given the heavy influence of the environment in the dynamic behavior of single residues. On the other hand, protein embeddings are often the byproduct of a machine or deep learning model using a protein sequence as input [12, 35], unlike the approach followed for ps3DDPDs. Here, instead, a common main framework was kept to increase the interpretability and interoperability of the resulting descriptors. This allowed us to follow a similar optimization route for both descriptor types (Fig. 2). In terms of residue composition, for our particular dataset the full sequence was favored. In a less diverse GPCR set, however, the use of family- or subfamily-specific alignments and binding pocket selections would provide more relevant information to the model given the differential activation-induced conformational changes reported for GPCRs binding different ligand types [21].

Next, the performance of our optimized 3DDPDs in PCM regression and classification tasks was tested using both random and temporal validation splits (Fig. 4). The performance of our models was in line with other PCM models trained in similar conditions for subfamilies of GPCRs [29]. In our set, 3DDPDs performed similarly to non-dynamic protein descriptors in classification tasks and regression tasks with a random split. These results suggest that the performance of these models had already reached its peak and small differences in the way to represent the protein space did not make a difference. Nevertheless, the best-performing models in classification tasks did not reach a high MCC. Models reached 0.646 ± 0.009 in the random split (Zscale van Westen), and 0.278 ± 0.005 in the temporal split (Zscale Hellberg), hence questioning the relevance of this dataset for such task. Interestingly, protein embeddings (UniRep) showed lower performance across the board, which has also been shown in other datasets compared to sequence- and 3D-based protein descriptors [36]. In the regression task with temporal split, however, 3DDPDs significantly outperformed non-dynamic descriptors. Given the more challenging form of validation introduced by the temporal split, the 3DDPDs represent an advantage. These results are likely also the result of performing 3DDPD optimization using this particular task. Nevertheless, similar behaviors have been observed in other benchmarks when using temporal splits compared to random splits [29, 37]. Moreover, in our PCM benchmark ps3DDPDs performed better than rs3DDPDs overall. One reason for this could be the difference in descriptor length: for the GPCR wild type set, rs3DDPDs contained 3785 features and ps3DDPDs 30 features. Moreover, the MSA used to compute rs3DDPD contained many gaps as it accounted for all class A GPCRs and not only the ones in the set. Therefore, lengthy rs3DDPDs with a large number of zeroes likely introduced noise in the model compared to the more compact ps3DDPDs. While this aspect would be corrected in practice by feature selection techniques prior to modelling, those were not applied here, similarly to hyperparameter optimization, to be able to explicitly benchmark the calculated descriptor with the least degrees of freedom. Finally, ps3DDPDs represent the overall differences between proteins in the set, which seems to be beneficial in agreement with the observation from Rackovsky and Scheraga that the description of the overall mobility of the protein correlates better with its structure than the description of individual residue mobility [38].

Subsequently, the biological relevance of the information contained in the 3DDPDs was investigated. One-hot encoding rs3DDPDs are calculated independently for each target and ps3DDPDs together for the targets in a particular set. Respectively, they exploit differences in atom coordinates and partial charges across positions in a target or a number of targets, representing the most relevant aspects of the protein dynamics, as defined by the RMSF fluctuations (Fig. 3, Additional file 2: Figs S1, S2, S3, S4). An advantage of rs3DDPDs is the possibility to be traced back to particular residues, alignment positions, or GPCR generic positions. This allowed us to investigate whether the 3DDPDs capture biologically relevant information from the MD simulation. To this end, the most important rs3DDPD features in regression PCM models were extracted and mapped to their corresponding GPCR generic positions (Fig. 6). The most important feature in a temporal split corresponded to the BW position 3.32 in TM3. As an example, in AA1R this translated to Val 87, which lies within the orthosteric binding pocket and makes hydrophobic interactions with the endogenous ligand adenosine (PDB 7LD4 [39]). Other important rs3DDPD features were located in the ECL2 and ECL3, which as expected showed high flexibility in the MD simulations and are regions whose conformational changes are known to be relevant for ligand binding [40] and activation [41]. In the models built with a random split, the two most important rs3DDPD features corresponded to TM1 1.38 and TM6 6.46 BW positions, respectively. In AA1R, these translated to Ile 15 and Leu 245, which flank the binding site of non-endogenous co-crystalized antagonists (PDB 5UEN [42]). The other two important rs3DDPD features correspond to positions in ICL3, which are close to the G protein interface (PDB 7LD3 [39]). These results confirm that 3DDPDs capture relevant changes for GPCR ligand binding and activation and could help elucidate functional sites in orphan proteins. Similar approaches have previously leveraged MD information to identify relevant functional sites using deep learning models [18] or graph-based approaches [43].

Finally, the use of 3DDPDs beyond wild type proteins was showcased by applying them to GPCR mutant MD simulations computed for a selection of 28 variants from five targets in our set with varied in vitro effects on ligand binding (Additional file 2: Fig S6). The analysis of the MD trajectories showed major dynamic fluctuations compared to wild type across the protein sequence, and not necessarily in the vicinity of the amino acid change, contrary to expectation (Additional file 2: Fig S7). Such allosteric effects on the protein dynamics dependent on the 3D organization of the protein have been previously shown to be able to explain the pathogenic mechanism of disease-driving variants [44, 45], as well as cancer mutational drivers [46], and are therefore relevant to encode. Since 3DDPDs could not be applied to predict mutant bioactivity due to the lack of available data for our set, the power of the dynamic descriptors to discriminate between variants was investigated by clustering them based on the distance between descriptor vectors. To this end, rs3DDPDs were able to cluster all variants of the same target together, and smaller clusters were formed for mutants with similar dynamic behaviors compared to the wild type (Fig. 7a, Additional file 2: Fig S7). Nevertheless, the clusters created based on rs3DDPDs did not fully represent the clusters based on RMSF (Additional file 2: Fig S8), further supporting that 3DDPDs include non-dynamic information on top of dynamic information. These results make us confident to propose the use of rs3DDPDs as mutant descriptors in machine learning tasks. Other works have highlighted the use of dynamic information to predict differences between mutants, such as by extracting normal modes [47], or time series of changing geometrical features [48]. However, as the changes in protein dynamics did not fully match the in vitro effects from the limited mutagenesis data available, the value in mutant bioactivity prediction needs to be further validated. Mutant clusters generated based on ps3DDPDs captured the most different dynamic changes between variants (Fig. 7b), but this did not result in the expected clustering. The biggest differences in RMSF between mutants were observed in the N- and C-terminus, which are the most flexible regions of the GPCR together with the loops. While the termini have a function in the receptor, in the context of ps3DDPDs it seems to be blown out of proportion. An alternative would be to compute ps3DDPDs for particular regions of interest. For instance, we suggest analyzing functionally relevant residues derived from rs3DDPD feature importance, from observations in the RMSF analysis, or the literature (for example for cancer-related mutants [24]).

One of the main limitations of our current approach is the reliability of MD simulations as input data for the computation of 3DDPDs. Firstly, the issue of MD stochastic stability is not addressed here [49], as different replicates are not used to compute our 3DDPDs. This was acceptable for the GPCR case study given the low inter-replicate variability found for MD simulations in GPCRmd. In the future, an analysis of the impact of additional replicates in the data collection phase should be conducted. The introduction of replicas could be done twofold, either by directly using the average of the atomic coordinates as starting point, or by using a bigger stack of individual atomic coordinates in the first PCA. Secondly, MD simulations are computationally expensive to generate, which can be a bottleneck. Similar publicly available repositories to those existing for GPCRs (i.e. GPCRmd) would help increase the applicability domain of dynamic descriptors to other protein families in the future. Finally, by extracting features from the MD trajectory, there is a constant need to make informed decisions to leave out data and reduce the amount of information available. Recently, graph neural networks (GNNs) have been used to represent MD trajectories [50]. The network embeddings could be used as dynamic descriptors instead, letting the machine decide which features are more relevant, although such approaches do not necessarily produce better results [51]. As a last note on applicability, in our current work the description of the dynamic behavior of a protein is tackled, but the conformational changes introduced by ligand binding are not taken into account. Running MD simulations for every complex in the dataset would not be advisable, but the dynamic binding space could be represented for example by an additional term describing dynamic pharmacophores [52] or computing cross-terms between dynamic protein and ligand descriptors [14].

## Conclusion

In this work, 3D dynamic protein descriptors (3DDPDs) were developed that capture the dynamic fluctuations of GPCRs as observed in MD simulations. Our one-hot encoding (rs3DDPDs) and embedding-like (ps3DDPDs) descriptors matched the performance in PCM tasks of non-dynamic state-of-the-art protein descriptors, outperforming them in regression tasks with a more challenging temporal split validation. Moreover, by mapping the most important rs3DDPD features in regression models to their GPCR generic positions it was shown that 3DDPDs represent biologically relevant information for ligand binding and activation. Finally, 3DDPDs were employed to discriminate mutant GPCRs based on their dynamic behavior with promising results that could be translated to the field of oncological drug discovery.

## Methods

### Wildtype GPCR MD trajectory selection and analysis

The MD simulations for the construction of 3D dynamic protein descriptors (3DDPDs) were obtained from GPCRmd [23] following the first official data deposit on November 14th 2019. Given the positive bias towards inactive conformations, apo simulations in inactive conformation were selected for class A GPCRs with available bioactivity data (see PCM bioactivity modelling). When more than one system was available PDB codes with true apo structure with the highest resolution were selected (Table 1). Most selected MD trajectories had been simulated in triplicate for 500 ns over 2500 frames following the GPCRmd standardized pipeline. The exceptions were GPCRmd ID 87 with 1250 frames and ID 154 with 2000 frames. For the generation of 3DDPDs, the first replicate was selected for each system.

**Table 1:** Wildtype GPCR MD trajectories selected from GPCRmd

GPCR	PDB	GPCRmd ID	Resolution (Å)
5HT1B	4IAR	87	2.80
5HT2B	4IB4	92	2.70
AA1R	5UEN	165	3.20
AA2AR	5IU4	49	1.72
ACM1	5CXV	154	2.70
ACM2	3UON	111	3.00
ACM4	5DSG	157	2.60
ADRB2	2RH1	11	2.40
AGTR1	4ZUD	189	2.80
CCR5	4MBS	118	2.71
CNR1	5U09	163	2.60
CXCR4	3ODU	101	2.50
DRD3	3PBL	105	2.89
EDNRB	5GLH	158	2.80
FFAR1	4PHU	75	2.33
HRH1	3RZE	108	3.10
LPAR1	4Z35	184	2.90
OPRD	4N6H	73	1.80
OPRK	4DJH	59	2.90
OPRX	5DHH	155	3.00
OX1R	4ZJ8	186	2.75
OX2R	4S0V	91	2.50
P2RY1	4XNV	179	2.20
P2Y12	4PXZ	77	2.50
PAR1	3VW7	128	2.20

Python library MDtraj [53] was used to compute the Root Mean Square Deviation (RMSD) and RMSF of MD trajectories to assess the stability of the simulations and account for differences in the dynamic behaviour of the selected GPCRs in different protein segments. RMSD was calculated for the protein atoms in reference to the first frame in the production run. RMSF was calculated for the protein Cα backbone atoms over the total length of the simulation. To allow direct comparison between receptors, RMSF values were aligned based on their corresponding residue number to the class A GPCR MSA obtained from GPCRdb [31]. The location of TM domains in the RMSF plots was mapped based on the generic BW [30] residue numbers obtained from GPCRdb. BW numbers were also used throughout the manuscript to refer to equivalent locations in the GPCR structure.

### 3DDPD generation and optimization

Atomic coordinates were extracted from GPCRmd trajectories with MDtraj. Each trajectory was divided into sub-trajectories of a defined number of frames, *f*, and the mean, median, and SD of the x, y, and z coordinates were calculated for each sub-trajectory. Additionally, atomic partial charges were generated for each atom in the system with RDkit Gasteiger charges calculator [54]. The next steps are tailored for the two flavors of 3DDPDs generated: one-hot encoding residue-specific (rs) 3DDPDs, and whole sequence embedding-like protein-specific (ps) 3DDPDs (Fig. 1).

For rs3DDPDs, coordinate statistics and partial charges per atom were collected for each target and standardized between 0 and 1. Subsequently, dimensionality reduction was applied in the form of PCA. A number of PCs for each atom were selected and grouped per residue as average and SD. A second dimensionality reduction step was applied to residue data and the selected PCs were placed in their matching sections corresponding to an MSA of the targets of interest.

Protein-specific ps3DDPDs were generated similarly to rs3DDPDs with some differences. Firstly, coordinate statistics and partial charges per atom were collected for all targets together and standardized between 0 and 1. Secondly, atom PCA was not grouped per residue and no second PCA was applied. Instead, the PCs selected per atom were grouped per target as average, median, and SD, constituting the final descriptor.

The generation parameters for the descriptors were randomly initialized and sequentially optimized. The parameters optimized included (in the following order):

i) Trajectory data: the use of all statistical values derived from the x, y, and z coordinates was compared to just the SD, representing the “rigidity” of each atomic coordinate.
ii) Frame split: number of frames included in each trajectory split, for which 10, 50, 100 and 500 frames were tested. This parameter was optimized on rs3DDPDs and the results were applied to ps3DDPDs.
iii) Residue PCA (only for rs3DDPDs): number of PCs selected after residue data PCA, either 3, 5, 7, or 10.
iv) Atom PCA coverage: variance explained by the selected number of PCs on atom data, either 95% or 99%.
v) Atom selection: inclusion of atomic data from all heavy atoms or just non-carbon atoms.
vi) Residue selection: strategies to focus the 3DDPDs on the protein binding site. These selections were based on structural-driven MSAs at different protein family levels, starting from using the full sequence, then the binding pocket of class A GPCRs, then of specific GPCR families, then GPCR subfamilies, and finally, target-specific binding pocket. To ensure a consistent number of features per descriptor, in rs3DDPDs only the first two options were tested.
vii) Combination with classical protein descriptors: tested sequentially and, for the case of rs3DDPDs also embedded on the descriptor via the residue PCA.

The optimization of 3DDPDs was done by comparing their performance with different parameters on PCM Bioactivity regression modelling on a temporal split.

### 3DDPD and MD hierarchical clustering

Hierarchical clustering dendrograms were computed to visualize similarities and differences between 3DDPD descriptors and dynamic behavior (represented by MD’s RMSF) across targets. Python package Scipy [55] was used to compute hierarchical clusters based on the Euclidean distance between non-null bits of 3DDPD or RMSF vectors. The accompanying representation of the descriptor or RMSF includes null bits that are derived from their mapping to the GPCR class A MSA. Plotting was done in Python using the package Matplotlib [56].

### PCM bioactivity modelling

The bioactivity dataset for PCM modelling was constructed starting from the highly curated Papyrus dataset version 5.50 [29]. For the regression task, high-quality datapoints with continuous data (pChEMBL values) were extracted for all available GPCRs. Receptors with MD inactive/intermediate apo trajectories available on GPCRmd and over 100 bioactivity datapoints were selected for the PCM set, resulting in 26 GPCRs and a total number of 38,701 bioactivity datapoints (Additional file 1: Table S1).

PCM modelling was implemented in Python 3.8 [57] using the modelling capabilities of the Papyrus scripts Python package [29]. Random Forest models from Scikit-learn [58] were used in regression and classification tasks as the state-of-the-art in bioactivity prediction. A pChEMBL value of 6.5 was considered as a cutoff between active and inactive compounds for classification tasks. Hyperparameters were set as default and not optimized during the training of the different models to reduce degrees of freedom in the comparison of the effect of different protein descriptors.

The compound descriptors used were Morgan fingerprints of radius 3 (ECFP6) and length 1024 [54], pre-calculated in the Papyrus dataset. The protein descriptors used to benchmark the performance of 3DDPDs were one-hot encodings and protein embeddings. The former included MS-WHIM, STscale, PhysChem, and two flavors of Zscale (Hellberg and van Westen, with 5 and 3 PCs per residue each) [10, 34]. One-hot encodings were calculated using the Python package ProDEC [59] based on the class A GPCR MSA obtained from GPCRdb for our protein set. As protein embeddings Unirep [60] were used, pre-calculated in the Papyrus dataset. 3DDPDs were benchmarked as protein descriptors on their own and in combination with non-dynamic protein descriptors. The best-performing rs3DDPDs and ps3DDPDs in the optimization phase were used for combination. Additionally, QSAR models were trained on each of the targets in the set with the same options and analysis as the PCM models to benchmark the use of protein descriptors.

Two methods were used to split the PCM dataset into training and test sets. Firstly a random split was used, where 80% of the data was allocated to the training set and 20% of the data to the test set. Data for all targets was present in both the training and the test set. Secondly, a temporal split was used to provide the model with a more challenging validation task than the random split, where compound-target pairs first recorded before 2013 were allocated to the training set, and newer datapoints to the test set. The cutoff year was selected to make sure that all targets were represented in the test set. This resulted in a test set with 39% of the data, which was not equally distributed per target but showed considerably reduced chemical bias between training and test set compared to the random split. Chemical bias was computed as the asymmetric validation embedding (AVE) bias defined by Wallach & Heifets [61] using as active-inactive cutoff a pChEMBL value of 6.5.

All RF models were trained using fivefold cross-validation, and the performance of the models was evaluated on the test set. The evaluation metrics reported were MCC for classification and Pearson *r* and RMSE for regression tasks. Other metrics are available in the additional data. For comparison purposes, a single average performance metric was calculated for QSAR RF models trained and tested on each target of the set independently.

Ten model replicates were trained for each protein descriptor benchmarked with random seeds 1234, 2345, 3456, 4567, 5678, 6879, 7890, 8901, 9012, and 9999. The seed was used for resampling, booth in the form of K-Fold shuffling in cross-validation and train/test splitting, the latter only in the case of a random split. Moreover, each model was initialized with a random seed as per default in Scikit-learn RF. The statistical significance of the differences in performance when using different protein descriptors was calculated by performing an independent T-test of the average performance metrics in the pool of model replicates. Differences were considered significant when p-value < 0.05. Performance comparison plots were generated in Python using the packages Matplotlib [56] and Seaborn [62].

### Selection of GPCR (cancer-related) somatic mutants

In order to test the usage of 3DDPDs in mutants, several mutations for the GPCRs in the 3DDPD set were selected. To simulate a real application scenario, a mutant PCM dataset was created, gathering available mutagenesis data from GPCRdb for the GPCR 3DDPD set. Mutations with datapoints available for more than ten different ligands were selected.

To extend the applicability domain, somatic mutations in cancer patients were extracted from the GDC database v22.0 [63] for the five GPCRs with selected mutagenesis data. Cancer-related mutations with mutagenesis data available on GPCRdb, regardless of the magnitude, were added to the mutation selection list in order to include a subsample of mutations present in cancer patients (Table 2).

**Table 2:** GPCR mutations selected

GPCR	PDB	GPCRmd ID	Mutation	GPCRdb ligands/datapoints	GDC patients	Motif
AA1R	5UEN	165	T277A^7.41^	13/36	0	–
			R291C^7.56^	4/4	1	NpxxY (ext)
			R296C^8.51^	4/4	1	–
AA2AR	5IU4	49	I66A^2.64^	20/22	0	–
			L85A^3.33^	21/21	0	–
			T88D^3.36^	14/16	0	–
			S91A^3.39^	12/16	0	–
			L167A^45.51^	20/20	0	–
			M177A^5.40^	22/24	0	–
			N181A^5.43^	20/20	0	–
			W246A^6.48^	37/52	0	CWxP
			N253A^6.55^	22/22	0	–
			Y271A^7.35^	20/22	0	–
			S277A^7.41^	29/33	0	–
			H278N^7.42^	3/3	1	–
ACM2	3UON	111	D103E^3.32^	32/42	0	–
			D103N^3.32^	12/15	0	–
			V421L^7.33^	1/1	1	–
ADRB2	2RH1	11	D79N^2.50^	12/12	0	–
			D130N^3.49^	11/11	0	DRY
			S203A^5.43^	12/12	0	–
			S204A^5.44^	13/13	0	–
			N293L^6.55^	12/12	0	–
			V317A^7.43^	5/5	1	–
CCR5	4MBS	118	Y108A^3.32^	12/20	0	–

### Mutant MD simulations and 3DDPDs

Mutant MD simulations were performed according to the GPCRmd pipeline [23]. Equilibrated GPCRmd wild type systems were obtained from the first frame of the first simulation replicate available online for the GPCRmd IDs defined in Table 1. Using the HTMD package [64], the mutations of interest were introduced and the systems were re-equilibrated using AceMD MD engine [65] and default GPCRmd parameters. Consecutively, the re-equilibrated trajectories were wrapped and 500ns production runs were simulated in triplicate with different random initialization seeds following the GPCRmd framework. Finally, the production trajectories were wrapped and rs3DDPDs and ps3DDPDs were generated from the first replicate.

### 3D visualization

Representations of proteins in 3D were generated using PyMOL 2.5.2 [66].

### Hardware

Mutant MD simulations were computed both on a local Rocky Linux 8 server and the Leiden University High-Performance Computing cluster ALICE. The local server contains Dual Xeon(R) E5-2650 v4 12 core CPU, 512 G DDR4 memory, 7 Nvidia GTX 1080/8 Gb mem, and 1 GeForce RTX 2080 Ti/11 Gb mem. MD simulations were computed on one GPU node each. PCM modelling and data analysis was done in the aforementioned local Rocky8 system.

### Supplementary Information

**Additional file 1:** Tables S1-S3.

**Additional file 2:** Figures S1-S8.

## Data Availability

Additional file 1: Tables S1–S3. Additional file 2: Figures S1–S8. The code used to generate and analyze these results can be accessed from https://doi.org/10.5281/zenodo.8026883 and is maintained at https://github.com/CDDLeiden/3ddpd. The data underlying the conclusions presented here is available at https://doi.org/10.5281/zenodo.7957235. Some preliminary results of this work were presented at the 12^th^ International Conference on Chemical Structures. The accompanying slide deck is available at https://doi.org/10.5281/zenodo.6772315.

## Abbreviations

3DDPD: Three-dimensional dynamic protein descriptor
BW: Ballesteros-Weinstein
ECL: Extracellular loop
GDC: Genomic data commons
GNN: Graph neural network
GPCR: G protein-coupled receptor
ICL: Intracellular loop
MCC: Matthews correlation coefficient
MD: Molecular dynamics
MSA: Multiple sequence alignment
NA: Non-applicable
PC: Principal component
PCA: Principal component analysis
PCM: Proteochemometric (modelling)
Ps3DDPD: Protein-specific 3DDPD
QSAR: Quantitative structure–activity relationship (modelling)
RF: Random forest
RMSD: Root mean square deviation
RMSE: Root mean square error
RMSF: Root mean square fluctuation
Rs3DDPD: Residue-specific 3DDPD
SD: Standard deviation
TM: Transmembrane (domain)
