# Supplementary material for: 3DDPDs: describing protein dynamics for proteochemometric bioactivity prediction. A case for (mutant) G protein-coupled receptors
Source: J Cheminform. 2023 Aug 28;15:74. doi: 10.1186/s13321-023-00745-5 (PMC10463931; doi:10.1186/s13321-023-00745-5)
Supplement: Supplementary file 1 — Additional file 1: Tables S1-S3. [file 13321_2023_745_MOESM1_ESM.pdf]

Additional file 1. Supplementary tables

Table S1. Papyrus bioactivity data distribution across the set of 26 wild type GPCRs.

		pchembl value (Mean)				
Target ID	Activity datapoints	Min	Max	Median	Mean	SD
P29274_WT	3991	4.00	11.00	6.82	6.88	1.17
P21554_WT	3741	4.00	10.52	6.82	6.91	1.20
P30542_WT	3519	4.00	12.20	6.48	6.58	1.01
P35462_WT	3152	3.10	10.54	7.62	7.49	1.17
P41145_WT	2910	4.09	11.52	6.85	7.02	1.41
P41143_WT	2219	4.00	10.74	7.00	6.89	1.37
P21453_WT	2038	4.03	10.80	7.82	7.66	1.46
O43614_WT	1901	4.30	10.05	6.91	6.85	1.17
O43613_WT	1820	4.19	9.80	6.09	6.34	1.11
O14842_WT	1304	4.16	9.52	6.60	6.53	0.92
P11229_WT	1273	4.03	10.85	6.50	6.67	1.20
P51681_WT	1252	4.04	11.52	7.28	7.13	1.41
P41146_WT	1155	4.32	10.43	7.54	7.51	1.08
P41595_WT	1125	4.19	9.96	6.69	6.75	0.87
P07550_WT	1002	3.85	10.92	7.68	7.53	1.54
Q9H244_WT	988	4.24	9.60	7.17	7.13	1.04
P30556_WT	876	4.01	10.00	5.23	5.90	1.72
P35367_WT	817	4.01	10.13	7.00	7.02	1.17
P08172_WT	791	4.02	10.36	6.92	6.98	1.30
P25116_WT	665	4.02	9.00	7.16	6.95	0.97
P08173_WT	584	4.00	10.75	6.41	6.49	1.03
P28222_WT	524	4.99	10.05	7.80	7.65	1.21
P61073_WT	402	4.15	9.21	7.04	6.91	0.94
P47900_WT	370	4.35	10.52	6.90	6.95	1.17
Q92633_WT	156	4.75	8.96	6.76	6.70	0.82
P24530_WT	126	4.00	9.39	6.01	6.08	1.00
Total	38,701					

Table S2. Performance metrics of QSAR and PCM models with random validation split trained with different protein descriptors. QSAR model performance represents the average over the individual target models trained and validated without protein descriptors (NA: non-applicable).

Model	Split	Protein descriptor	Metric	mean	std
QSAR	random	NA	MCC	0.577714	0.007181
QSAR	random	NA	RMSE	0.705380	0.005661
QSAR	random	NA	r	0.774895	0.004677
QSAR	random	NA	R2	0.601279	0.007420
QSAR	random	NA	MAE	0.523834	0.004150
PCM	random	3DDPD_PS_all_f100_pc95_fs_aa	MCC	0.644716	0.00675
PCM	random	3DDPD_PS_all_f100_pc95_fs_aa	RMSE	0.704416	0.006187
PCM	random	3DDPD_PS_all_f100_pc95_fs_aa	r	0.835304	0.002997
PCM	random	3DDPD_PS_all_f100_pc95_fs_aa	R2	0.693622	0.004761
PCM	random	3DDPD_PS_all_f100_pc95_fs_aa	MAE	0.527470	0.004374
PCM	random	3DDPD_RS_std_f100_pc10_fs_aa	MCC	0.642643	0.008263
PCM	random	3DDPD_RS_std_f100_pc10_fs_aa	RMSE	0.710025	0.006935
PCM	random	3DDPD_RS_std_f100_pc10_fs_aa	r	0.832272	0.003893
PCM	random	3DDPD_RS_std_f100_pc10_fs_aa	R2	0.688707	0.006327
PCM	random	3DDPD_RS_std_f100_pc10_fs_aa	MAE	0.531428	0.005215
PCM	random	MS-WHIM	MCC	0.645082	0.007837
PCM	random	MS-WHIM	RMSE	0.706699	0.00666
PCM	random	MS-WHIM	r	0.834099	0.003879
PCM	random	MS-WHIM	R2	0.691614	0.006109
PCM	random	MS-WHIM	MAE	0.529213	0.004668
PCM	random	PhysChem	MCC	0.643758	0.00737
PCM	random	PhysChem	RMSE	0.707704	0.005797
PCM	random	PhysChem	r	0.833691	0.003228
PCM	random	PhysChem	R2	0.690748	0.005079
PCM	random	PhysChem	MAE	0.530157	0.004242
PCM	random	STscale	MCC	0.642903	0.007576
PCM	random	STscale	RMSE	0.706985	0.006782
PCM	random	STscale	r	0.834184	0.002843
PCM	random	STscale	R2	0.691390	0.004603
PCM	random	STscale	MAE	0.529546	0.005007
PCM	random	Zscale_Hellberg	MCC	0.644947	0.006025
PCM	random	Zscale_Hellberg	RMSE	0.706032	0.007275
PCM	random	Zscale_Hellberg	r	0.834601	0.004027
PCM	random	Zscale_Hellberg	R2	0.692197	0.006316
PCM	random	Zscale_Hellberg	MAE	0.529160	0.004882
PCM	random	Zscale_van_Westen	MCC	0.645763	0.008597
PCM	random	Zscale_van_Westen	RMSE	0.703212	0.006583
PCM	random	Zscale_van_Westen	r	0.836407	0.003532

PCM	random	Zscale_van_Westen	R2	0.694661	0.005435
PCM	random	Zscale_van_Westen	MAE	0.527693	0.004787
PCM	random	unirep	MCC	0.642587	0.004844
PCM	random	unirep	RMSE	0.710875	0.006126
PCM	random	unirep	r	0.831989	0.003264
PCM	random	unirep	R2	0.687973	0.005269
PCM	random	unirep	MAE	0.532397	0.004853

Table S3. Performance metrics of QSAR and PCM models with temporal validation split trained with different protein descriptors. QSAR model performance represents the average over the individual target models trained and validated without protein descriptors (NA: non-applicable).

**Model**	**Split**	**Protein descriptor**	**Metric**	**mean**	**std**
QSAR	temporal	NA	MCC	0.191889	0.009093
QSAR	temporal	NA	RMSE	1.168438	0.003839
QSAR	temporal	NA	r	0.343006	0.004332
QSAR	temporal	NA	R2	-0.171235	0.008062
QSAR	temporal	NA	MAE	0.931834	0.002768
PCM	temporal	3DDPD_PS_all_f100_pc95_fs_aa	MCC	0.277186	0.00761
PCM	temporal	3DDPD_PS_all_f100_pc95_fs_aa	RMSE	1.153923	0.002905
PCM	temporal	3DDPD_PS_all_f100_pc95_fs_aa	r	0.451019	0.003336
PCM	temporal	3DDPD_PS_all_f100_pc95_fs_aa	R2	0.154453	0.004147
PCM	temporal	3DDPD_PS_all_f100_pc95_fs_aa	MAE	0.919372	0.002084
PCM	temporal	3DDPD_RS_std_f100_pc10_fs_aa	MCC	0.273142	0.003223
PCM	temporal	3DDPD_RS_std_f100_pc10_fs_aa	RMSE	1.213864	0.005166
PCM	temporal	3DDPD_RS_std_f100_pc10_fs_aa	r	0.41746	0.003671
PCM	temporal	3DDPD_RS_std_f100_pc10_fs_aa	R2	0.064317	0.007955
PCM	temporal	3DDPD_RS_std_f100_pc10_fs_aa	MAE	0.954875	0.003743
PCM	temporal	MS-WHIM	MCC	0.276817	0.003861
PCM	temporal	MS-WHIM	RMSE	1.218501	0.004445
PCM	temporal	MS-WHIM	r	0.410101	0.004479
PCM	temporal	MS-WHIM	R2	0.057159	0.006687
PCM	temporal	MS-WHIM	MAE	0.959843	0.003392
PCM	temporal	PhysChem	MCC	0.27533	0.004877
PCM	temporal	PhysChem	RMSE	1.21395	0.005249
PCM	temporal	PhysChem	r	0.414679	0.003797
PCM	temporal	PhysChem	R2	0.064184	0.007884
PCM	temporal	PhysChem	MAE	0.958551	0.003209
PCM	temporal	STscale	MCC	0.277505	0.004956
PCM	temporal	STscale	RMSE	1.217626	0.007125
PCM	temporal	STscale	r	0.413211	0.004509

PCM	temporal	STscale	R2	0.058495	0.010720
PCM	temporal	STscale	MAE	0.960353	0.004845
PCM	temporal	Zscale_Hellberg	MCC	0.278328	0.005163
PCM	temporal	Zscale_Hellberg	RMSE	1.22066	0.003245
PCM	temporal	Zscale_Hellberg	r	0.409729	0.003008
PCM	temporal	Zscale_Hellberg	R2	0.053820	0.004890
PCM	temporal	Zscale_Hellberg	MAE	0.962210	0.001808
PCM	temporal	Zscale_van_Westen	MCC	0.274272	0.008416
PCM	temporal	Zscale_van_Westen	RMSE	1.221101	0.006088
PCM	temporal	Zscale_van_Westen	r	0.409944	0.005973
PCM	temporal	Zscale_van_Westen	R2	0.053121	0.009200
PCM	temporal	Zscale_van_Westen	MAE	0.962816	0.004110
PCM	temporal	unirep	MCC	0.273962	0.00843
PCM	temporal	unirep	RMSE	1.219178	0.004602
PCM	temporal	unirep	r	0.411132	0.003577
PCM	temporal	unirep	R2	0.056110	0.007120
PCM	temporal	unirep	MAE	0.959212	0.003379
