# Supplementary material for: 3DDPDs: describing protein dynamics for proteochemometric bioactivity prediction. A case for (mutant) G protein-coupled receptors
Source: J Cheminform. 2023 Aug 28;15:74. doi: 10.1186/s13321-023-00745-5 (PMC10463931; doi:10.1186/s13321-023-00745-5)
Supplement: Supplementary file 2 — Additional file 2: Figures S1-S8. [file 13321_2023_745_MOESM2_ESM.pdf]

Additional file 2. Supplementary figures

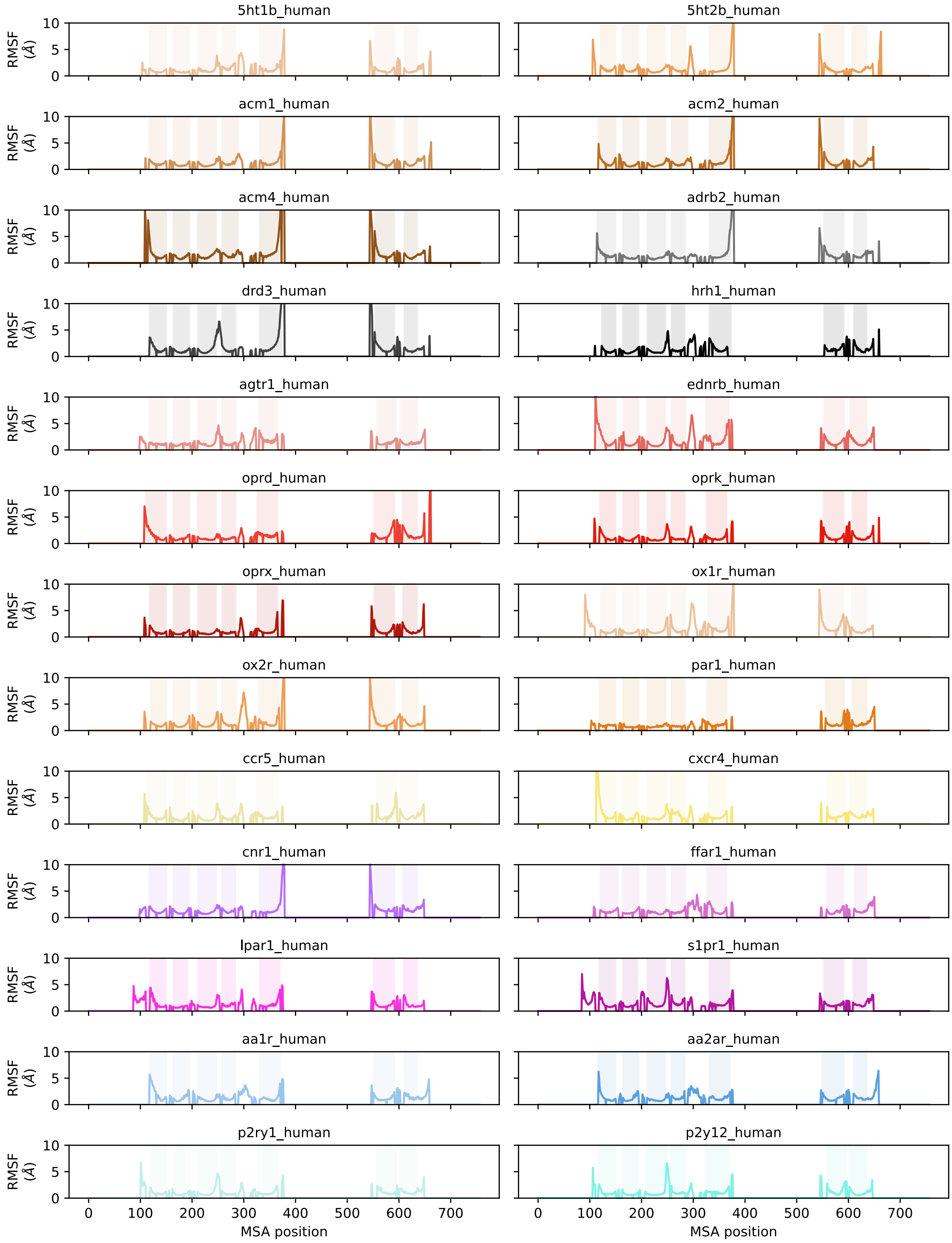

Figure S1. RMSF values for the MD GPCRmd trajectories of the 26 GPCRs in the wild type set. *The RMSF values are mapped to their corresponding positions in the MSA later used for rs3DDPD and non-dynamic descriptor calculation, for easier visualization. The regions in the MSA corresponding to domains TM 1-7 are shadowed for reference. Each receptor is represented in a different color and receptors from the same subfamily/family are represented in the same color palette.*

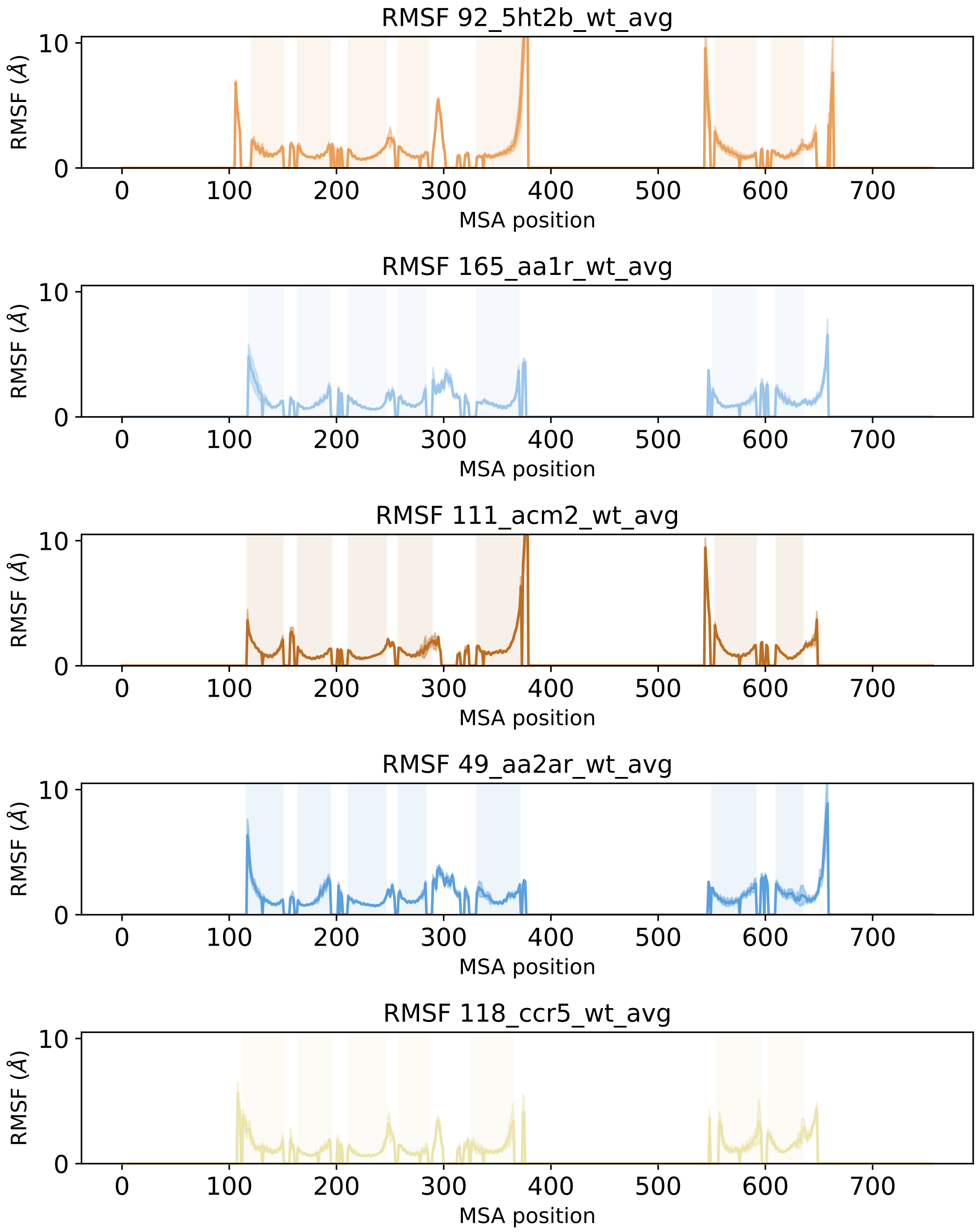

Figure S2. RMSF average and variability over the three GPCRmd trajectory replicates for GPCRs 5HT2B, AA1R, ACM2, AA2AR, and CCR5. *The average RMSF is represented as a line and the standard deviation of the mean is represented as a shade around the average. For easier comparison between targets, RMSF is aligned to the reference MSA and the transmembrane domains TM1-7 are shaded.*

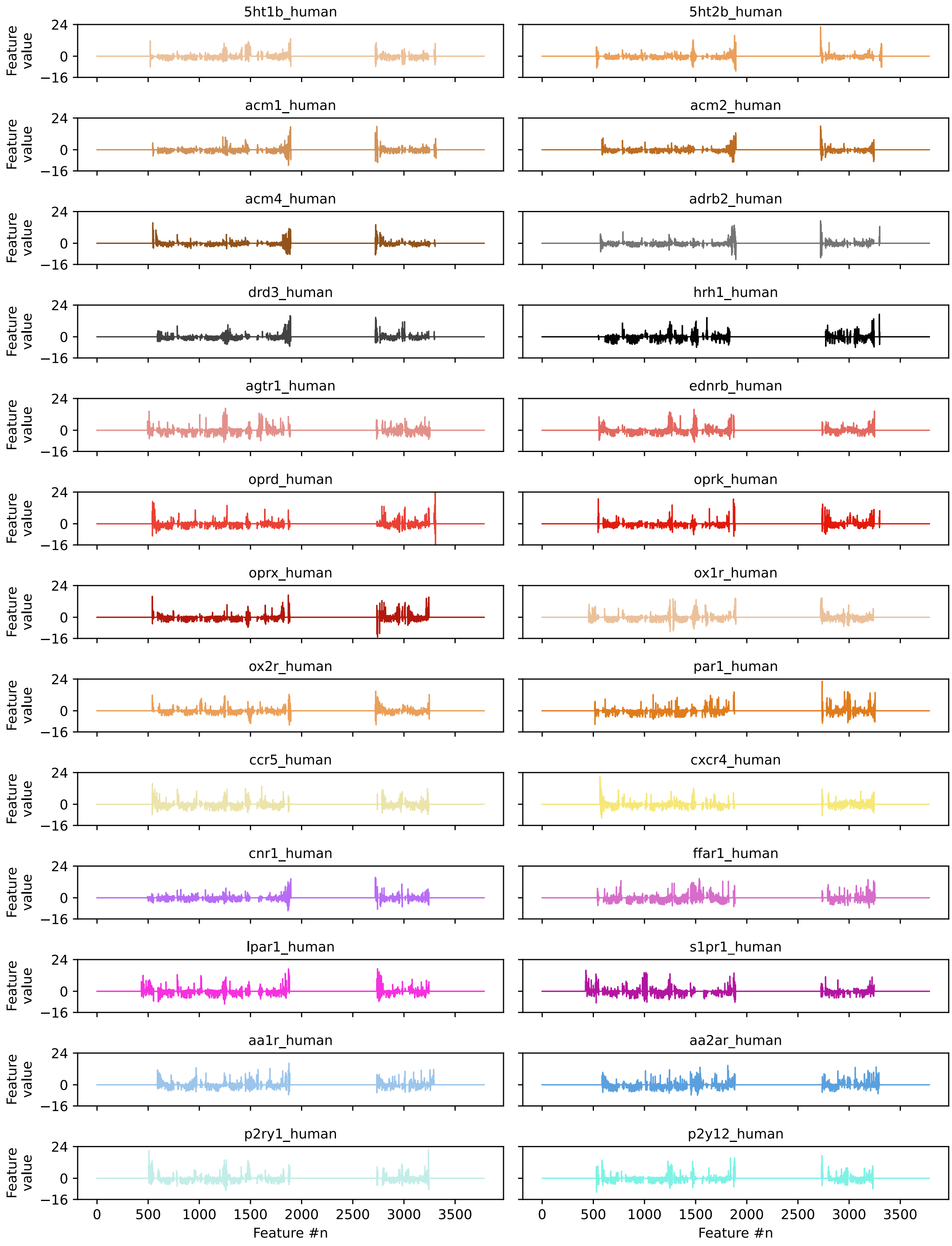

Figure S3. Representation of rs3DDPD feature values for the 26 GPCRs in the wild type set. *Each receptor is represented in a different color and receptors from the same subfamily/family are represented in the same color palette.*

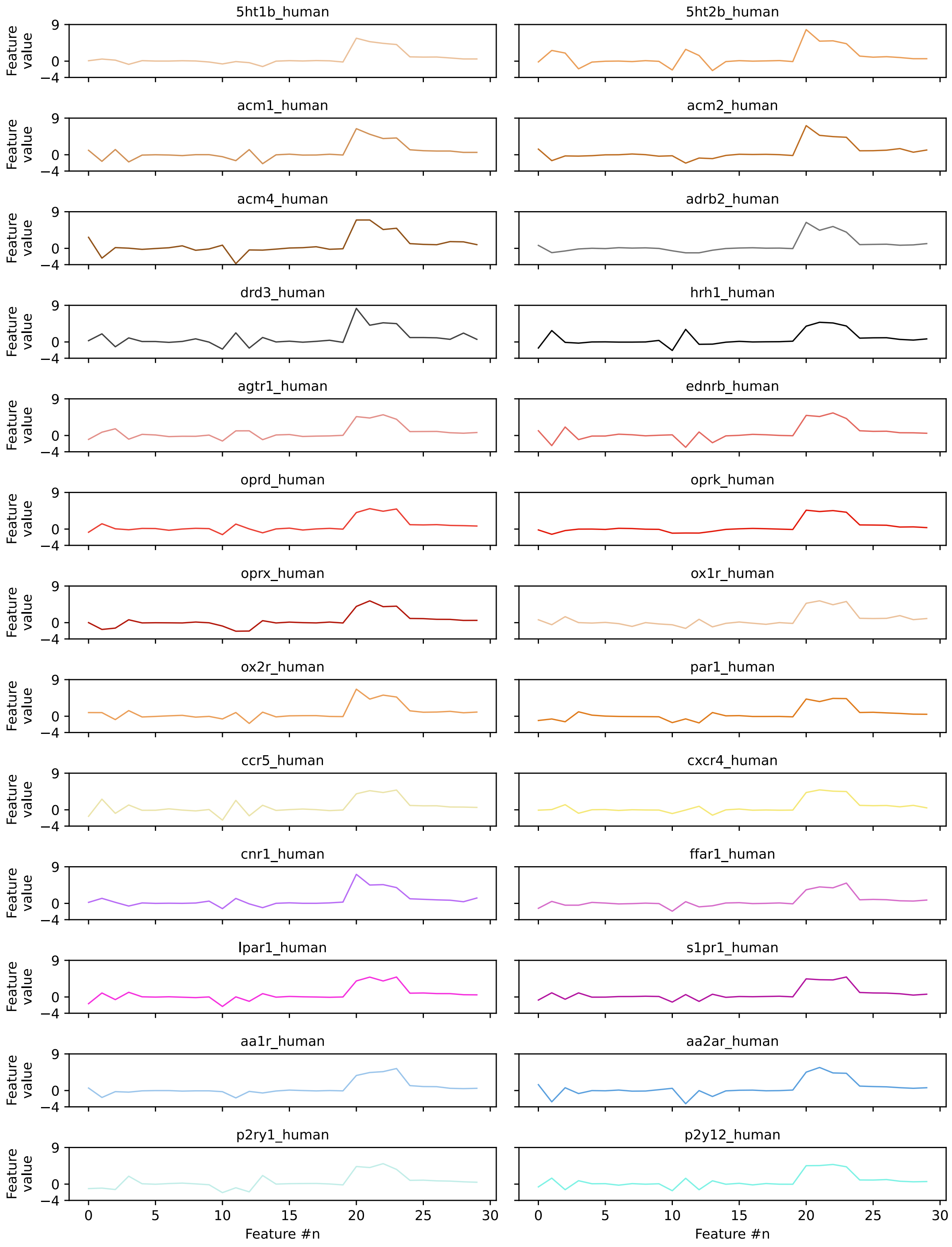

Figure S4. Representation of ps3DDPD feature values for the 26 GPCRs in the wild type set. *Each receptor is represented in a different color and receptors from the same subfamily/family are represented in the same color palette.*

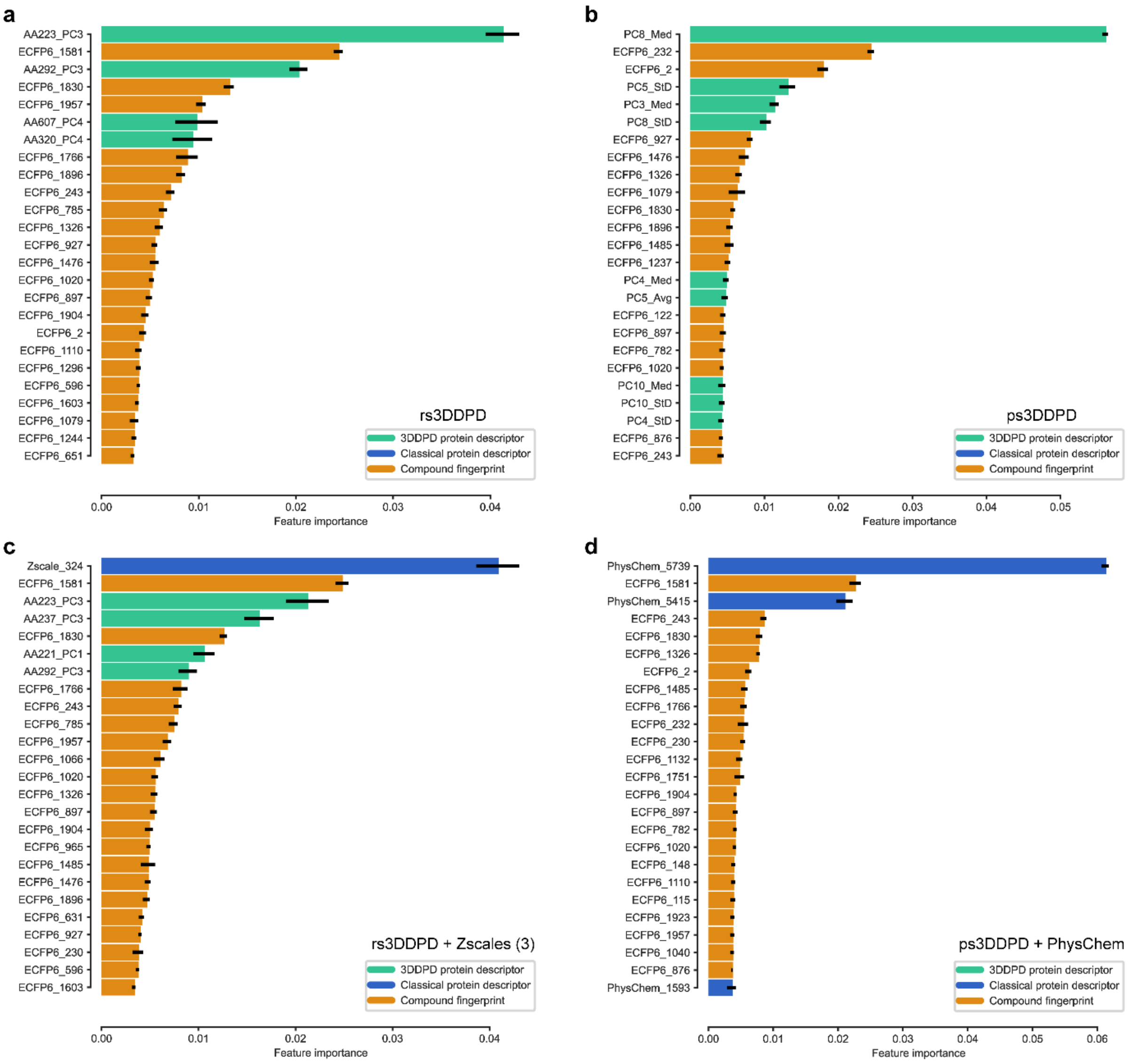

Figure S5. Top 25 most important features in PCM regression models using a temporal split validation. *The importance was averaged across the ten random seeds trained and the SD represented as error bars. The models were trained on the following protein descriptors:* ***(a)*** *rs3DDPD,* ***(b)*** *ps3DDPD,* ***(c)*** *combination of rs3DDPD and Zscale van Westen,* ***(d)*** *combination of ps3DDPD and PhysChem.*

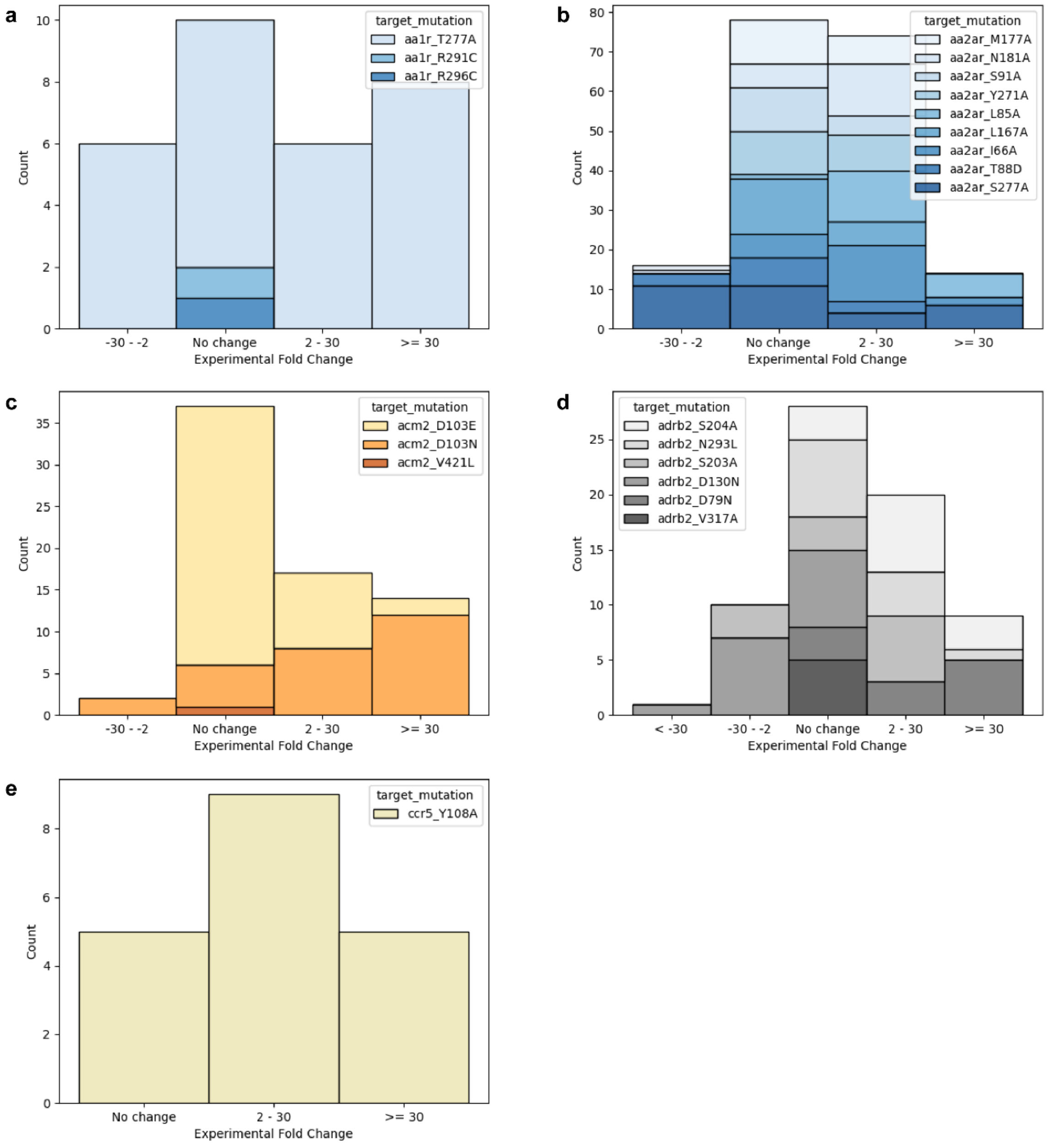

Figure S6. Distribution of* in vitro *consequences from available mutagenesis data for the GPCR mutant set in GPCRdb. *In the y axis it is represented the amount of ligands with available experimental fold change of virtually no change (between -2 and 2), positive or negative change (between absolute 2 and 30 fold change), or big positive or negative change (bigger than absolute 30 fold change). Bars are stacked for each mutant of the five targets in the set:* ***(a)*** *adenosine A1 receptor (AA1R),* ***(b)*** *adenosine A2A receptor (AA2AR),* ***(c)*** *muscarinic acetylcholine receptor 2 (ACM2),* ***(d)*** *beta-2 adrenergic receptor (ADRB2),* ***(e)*** *CC chemokine receptor 5 (CCR5).*

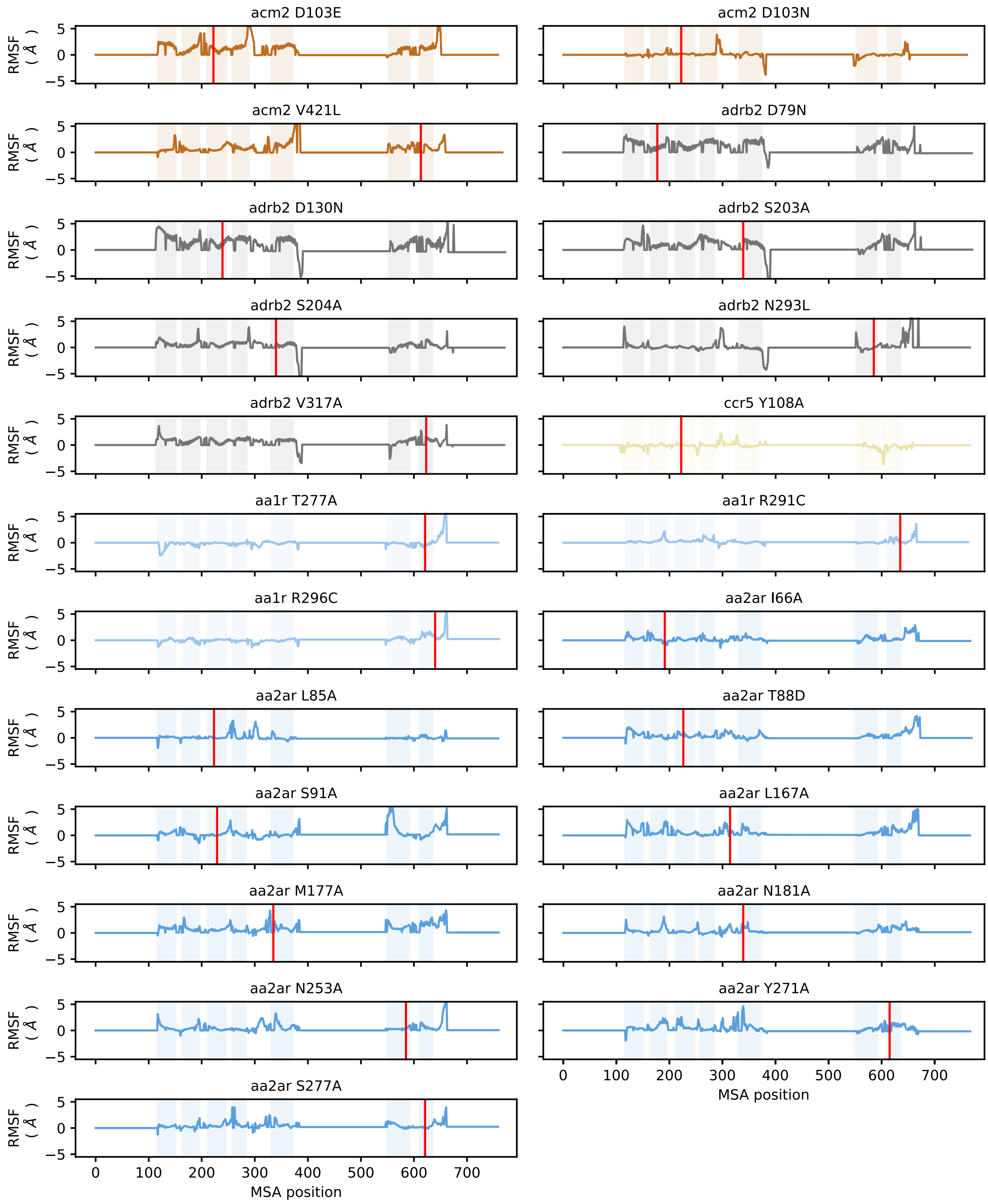

__Figure S7. Mutant GPCR RMSF normalized to wild type.__ RMSF values are aligned to the MSA for easier comparison between targets. Domains representing TM 1-7 are shadowed. The location of the mutation in the MSA is highlighted in red.

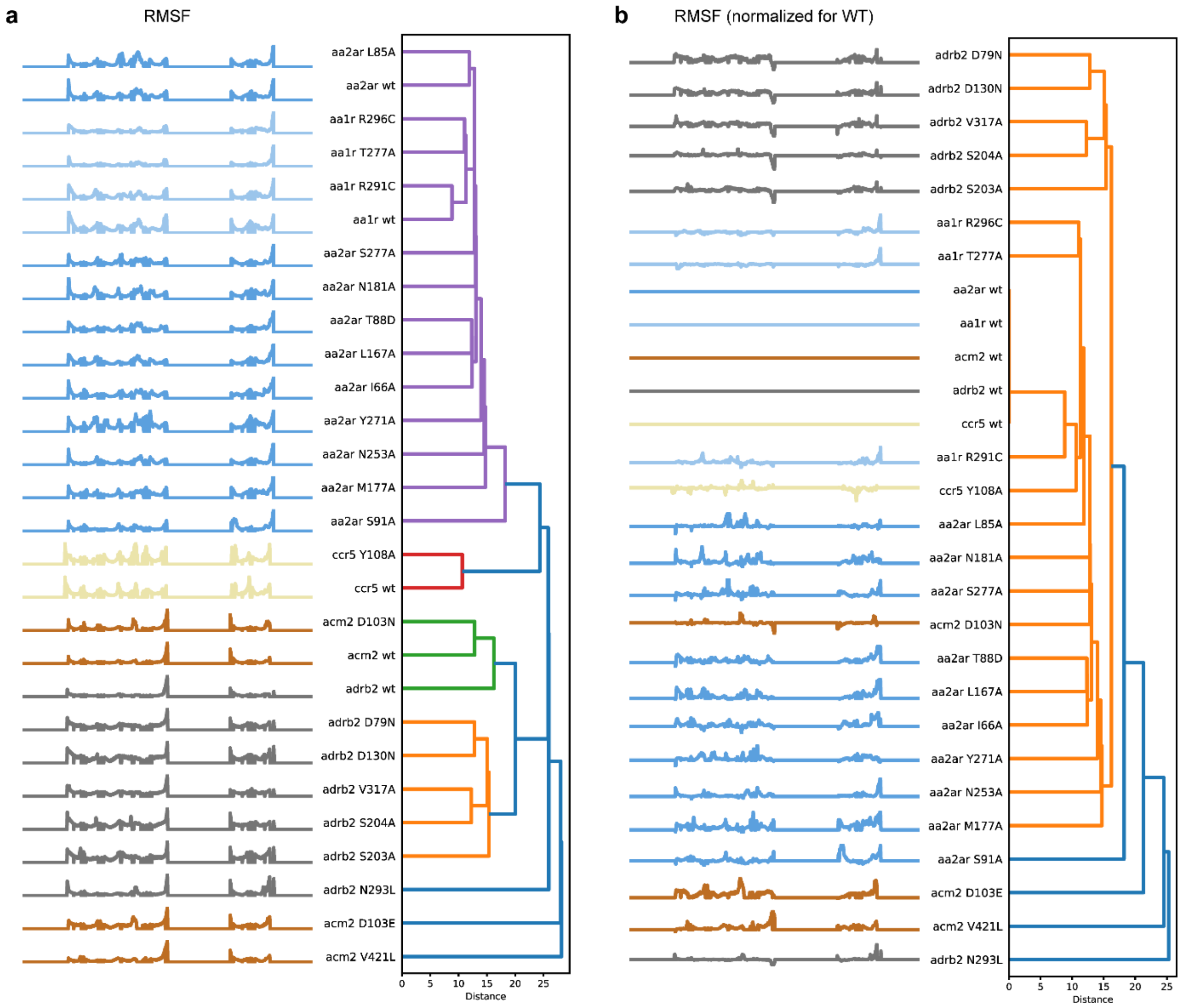

Figure S8. Discrimination of GPCR mutants using RMSF. *Hierarchical clustering of GPCR variants based on their Euclidean distance between RMSF vectors.* ***(a)*** *Mutants represented as MSA-aligned RMSF.* ***(b)*** *Mutants represented as MSA-aligned normalized to wild type. Individual clusters generated under a distance threshold of 70 % of the final merge are represented in different colors in the dendrograms.*
